# DNA damage promotes ER stress resistance through elevation of unsaturated phosphatidylcholine in *Caenorhabditis elegans*

**DOI:** 10.1074/jbc.RA120.016083

**Published:** 2020-11-24

**Authors:** Jianhui Deng, Xue Bai, Haiqing Tang, Shanshan Pang

**Affiliations:** School of Life Sciences, Chongqing University, Chongqing, China

**Keywords:** DNA damage response, ER stress response, apoptotic genes, fatty acid, phosphatidylcholine, *C. elegans*, SKN-1, BH3, Bcl-2 homology 3, DTT, dithiothreitol, ER, endoplasmic reticulum, FA, fatty acid, FAMEs, FA methyl esters, IRE-1, inositol-requiring enzyme 1, NGM, nematode growth medium, OA, oleic acid, PC, phosphatidylcholine, PE, phosphatidylethanolamine, RNAi, RNA interference, SFAs, saturated FAs, SKN-1, skinhead-1, SREBPs, sterol regulatory element-binding proteins, UFAs, unsaturated FAs, UPR^ER^, unfolded protein response of the endoplasmic reticulum, UPR^mt^, mitochondria-specific UPR, XBP-1, X-box binding protein 1

## Abstract

DNA damage triggers the cellular adaptive response to arrest proliferation and repair DNA damage; when damage is too severe to be repaired, apoptosis is initiated to prevent the spread of genomic insults. However, how cells endure DNA damage to maintain cell function remains largely unexplored. By using *Caenorhabditis elegans* as a model, we report that DNA damage elicits cell maintenance programs, including the unfolded protein response of the endoplasmic reticulum (UPR^ER^). Mechanistically, sublethal DNA damage unexpectedly suppresses apoptotic genes in *C. elegans*, which in turn increases the activity of the inositol-requiring enzyme 1/X-box binding protein 1 (IRE-1/XBP-1) branch of the UPR^ER^ by elevating unsaturated phosphatidylcholine. In addition, UPR^ER^ activation requires silencing of the lipid regulator skinhead-1 (SKN-1). DNA damage suppresses SKN-1 activity to increase unsaturated phosphatidylcholine and activate UPR^ER^. These findings reveal the UPR^ER^ activation as an organismal adaptive response that is important to maintain cell function during DNA damage.

Accumulation of DNA damage is a crucial driving force for animal aging and several human diseases. Cells have evolved multiple elegant programs to respond to DNA damage and maintain cellular homeostasis. DNA repair machinery is one such program that detects and fixes DNA damage with a series of defined enzymes. However, when DNA damage is too severe to be fully repaired, which is detrimental to cell function, the cells would initiate apoptosis to terminate the damage spread (1). During DNA damage, cellular maintenance is critical as it may facilitate endurance to allow DNA repair and ensure cell function. Yet whether and how cellular maintenance program is regulated by DNA damage is not fully explored.

*Caenorhabditis elegans* is an ideal model for DNA damage response study because of its genetic tractability. The DNA damage responses have been explored in the germ cells of *C. elegans* (2), whereas the organismal adaptation to DNA damage is only beginning to be understood. Accumulation of DNA damage is deleterious to organismal health. DNA pair capacity declines with age in *C. elegans* and is required for normal life span after UV-induced DNA damage (3, 4). Accordingly, adaptive responses are induced to counteract DNA damage in *C. elegans*. Longevity factor abnormal dauer formation-16/forkhead box O (DAF-16/FoxO) was found to be required for alleviating developmental arrest and antagonizing aging driven by persistent DNA damage (5), implying that a survival-boosting program is involved in DNA damage response in *C. elegans*. In addition, transcriptomic, proteomic, and lipidomic analyses revealed that persistent DNA damage in *C. elegans* correlates with several cellular responses, such as autophagy activation and fatty acid (FA) reduction (6).

In the present study, we explored the regulation of cellular maintenance program by DNA damage in *C. elegans* and found that severe DNA damage promotes endoplasmic reticulum (ER) and mitochondrial stress resistance. Intriguingly, the apoptotic pathway genes were suppressed by DNA damage simultaneously to favor cell preservation. These two responses are intimately integrated: the induction of the unfolded protein response of the endoplasmic reticulum (UPR^ER^) requires the suppression of apoptotic genes. Furthermore, we found that DNA damage increases the activity of the UPR^ER^ via unsaturated phosphatidylcholine (PC), which is antagonized by the transcription factor skinhead-1 (SKN-1). Our findings reveal survival-boosting responses to severe DNA damage in *C. elegans*, which might be conserved during evolution.

## Results

### DNA damage promotes stress resistance in *C. elegans*

**Figure 1:**
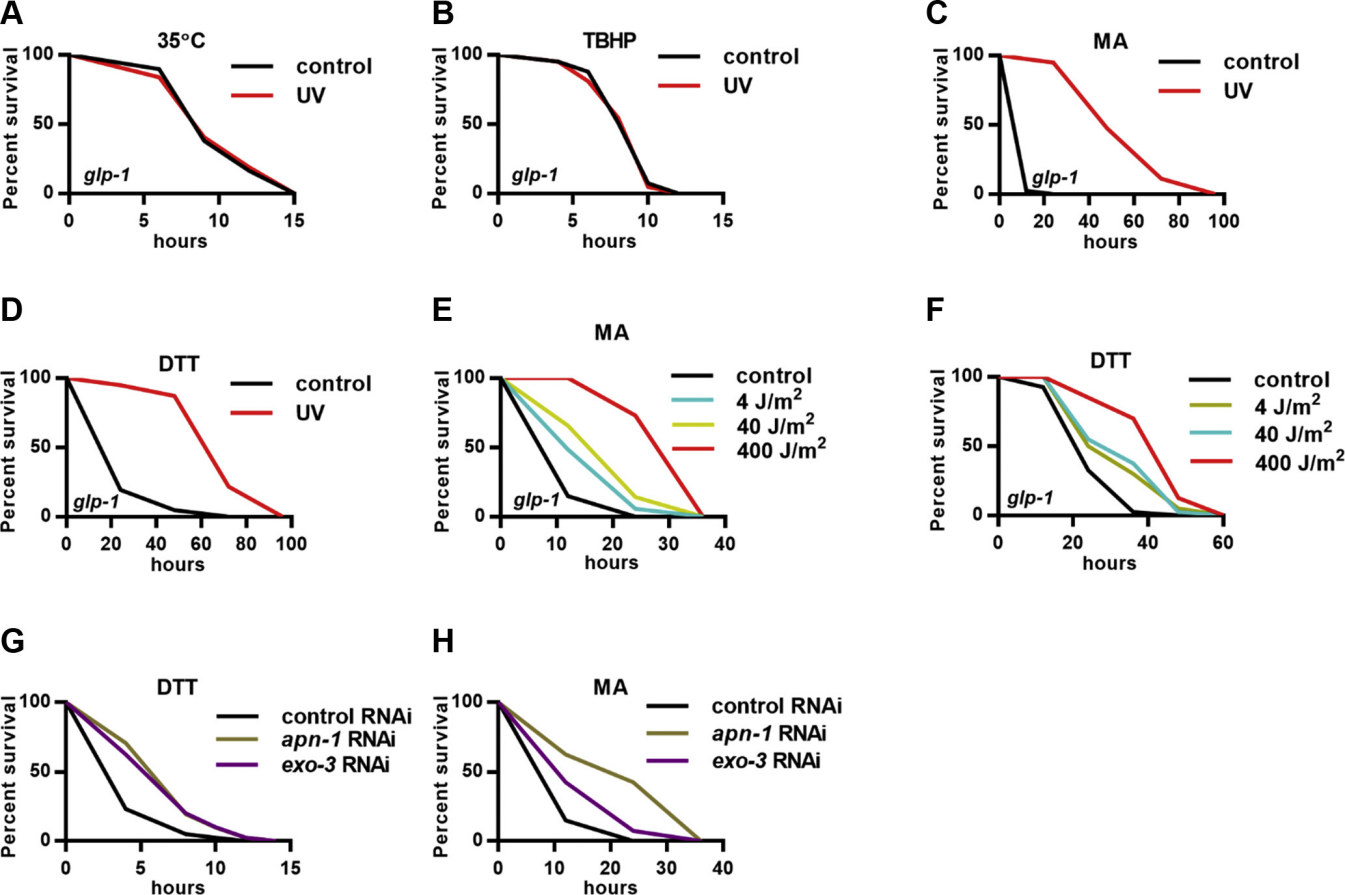
**DNA damage in somatic cells promotes stress resistance in germ line–deficient *C. elegans*.***A*–*D*, survival of UV-treated *glp-1* mutants in response to 35 °C heat shock (*A*), TBHP-induced oxidative stress (*B*), MA-induced mitochondrial stress (*C*), and DTT-induced endoplasmic reticulum stress (*D*). *E*–*F*, dose-dependent effects of UV exposure on MA (*E*) and DTT (*F*) resistance in *glp-1* mutants. *G*–*H*, the intestine-specific RNAi of *apn-1* and *exo-3* promotes resistance to (*G*) DTT and (*H*) MA in germ line–deficient *C. elegans*. DTT, dithiothreitol; MA, malonic acid; RNAi, RNA interference; TBHP, tert-butyl hydroperoxide.

We mainly used germ line–deficient *C. elegans* for the present study, as the somatic cells are highly resistant to DNA damage–induced cell death (7, 8), which are suitable for studying the adaptive response on DNA damage when cell death is not induced. Abnormal germ line proliferation-1/Notch is essential for germ line stem cell proliferation. The temperature-sensitive *glp-1* mutant *C. elegans* lack the germ line when growing at the restrictive temperature of 25 °C (9). Previous studies have reported that a UV-C dose of 50 to 150 J/m^2^ could induce DNA damage and shorten life span in wild-type *C. elegans* (3, 4, 10, 11, 12). We exposed day 1 adult *glp-1* worms to UV-C radiation of 400 J/m^2^, known to dramatically cause DNA damage in *glp-1* mutants (3, 4). Consistently, we found that this dose of UV-C significantly reduced somatic life span (Fig. S1*A*, Table S1), confirming life-threatening genotoxic stress in the soma. Because stress responses are crucial for cell maintenance and survival, we assessed animal responses to several stresses, including heat stress, oxidative stress, mitochondrial stress, and ER stress. The results showed that UV exposure had no effects on heat stress and oxidative stress resistance (Fig. 1, *A*–*B*, Table S1), whereas dramatically enhanced animal resistance to mitochondrial stress and ER stress in *glp-1* mutants (Fig. 1, *C*–*D*, Table S1). Similar effects were observed in another germ line–deficient model *glp-4* mutants (Fig. S1, *B*–*E*, Table S1). In addition, UV promoted mitochondrial and ER stress resistance in a dose-dependent manner (Fig. 1, *E*–*F*, Table S1). These observations suggest that DNA damage can initiate adaptive and beneficial responses that support cell maintenance. In addition, we found that germ line–intact wild-type animals that were treated with UV showed improved resistance not only to mitochondrial and ER stresses but also to heat and oxidative stresses (Fig. S1, *F*–*I*, Table S1), consistent with a previous finding demonstrating that DNA damage in dividing germ cells can promote somatic heat and oxidative stress resistance (13). It should be noted that the proliferating germ cells may also respond to DNA damage that in turn modulate mitochondrial and ER stress resistance in the soma of wild-type animal cells nonautonomously.

Cells initiate DNA repair pathways in response to DNA damage. Defects in the repair pathways would result in endogenous DNA damage even in the absence of exogenous genomic insults. Base excision repair pathway is one such pathway that is initiated by DNA glycosylase to excise the damaged base and form the apurinic/apyrimidinic (AP) site, which is then cleaved by AP endonuclease and further processed to finish the repair process. Knockdown of the *C. elegans* AP endonuclease gene increases the accumulation of spontaneous mutations in the soma (12). To understand whether endogenous DNA damage in somatic cells could also promote stress resistance, we knocked down *apn-1* and *exo*-*3*, two *C. elegans* AP endonuclease genes, specifically in the intestine and found that these treatments led to the enhanced resistance to ER and mitochondrial stress (Fig. 1, *G*–*H*, Table S1), phenocoping the UV-exposed animals. In addition, manipulations of *apn-1* or *exo-3* only modestly increased oxidative stress resistance (Fig. S1*J*, Table S1) and had no effects on heat stress resistance (Fig. S1*K*, Table S1). Thus, *C. elegans* appear to monitor multiple forms of DNA damages and mount cellular maintenance responses, characterized by the improved resistance to mitochondrial stress and ER stress.

### DNA damage activates the IRE-1/XBP-1 branch of the UPR^ER^

**Figure 2:**
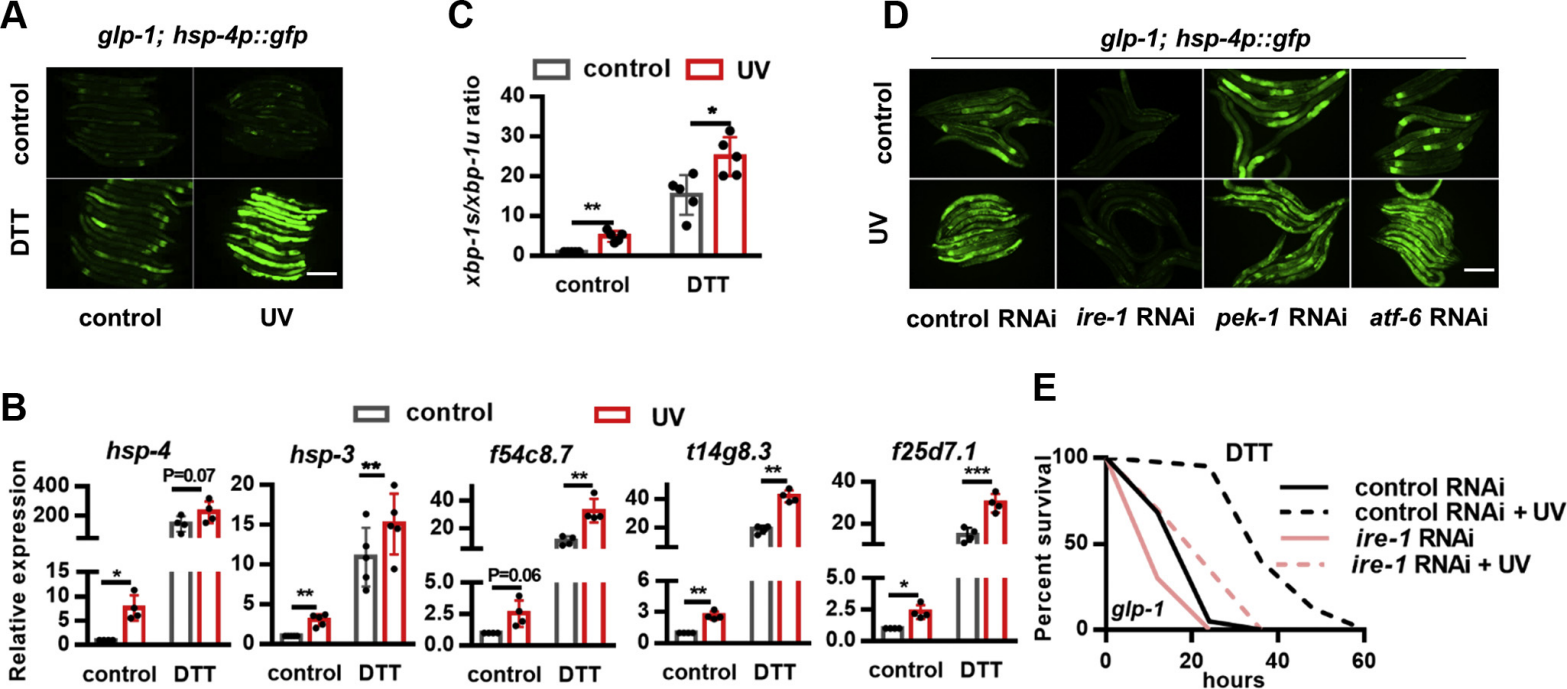
**DNA damage promotes the endoplasmic reticulum unfolded protein response.***A*–*C*, UV-exposed *glp-1* mutants show elevated expression of *hsp-4p*::GFP (*A*), increased mRNA levels of XBP-1 target genes (*B*), and increased ratio of *xbp-1s* to *xbp-1u* (*C*) in response to DTT. *D*, effects of *ire-1*, *pek-1*, and *atf-6* RNAi on UV-induced *hsp-4p*::GFP expression in response to DTT. *E*, effects of *ire-1* RNAi on UV-induced endoplasmic reticulum stress resistance. ∗*p* < 0.05, ∗∗*p* < 0.01, ∗∗∗*p* < 0.001. The scale bar represents 100 μm. DTT, dithiothreitol; RNAi, RNA interference.

The induction of stress resistance is associated with the increased expression of stress response genes. Mitochondrial stress activates the mitochondria-specific UPR (UPR^mt^) that is characterized by the elevated expression of the mitochondrial chaperone *hsp-6* (14). However, the expression of *hsp-6p*::GFP, induced by antimycin, an inhibitor of mitochondrial respiration, was not enhanced by UV exposure (Fig. S2*A*). ER stress induces the UPR^ER^ characterized by the enhanced expression of the ER chaperone *hsp-4* (heat shock protein-4) (15). The basal expression of *hsp-4p*::GFP reporter is very low, but when exposed to UV, the *glp-1* mutants showed enhanced expression of *hsp-4p*::GFP in response to either dithiothreitol (DTT) (Fig. 2*A*) or tunicamycin (Fig. S2*B*), two chemicals specific for ER stress induction. Quantitative PCR analysis validated that the basal as well as the DTT-induced expression of *hsp-4* was promoted by UV exposure (Fig. 2*B*). Wild-type worms showed similar induction of *hsp-4p*::GFP on UV exposure (Fig. S2*C*). In addition, the RNA interference (RNAi) knockdown of either *apn-1* or *exo-3* also modestly increased the expression of *hsp-4p*::GFP in response to ER stress (Fig. S2*D*).

The inositol-requiring enzyme 1 (IRE-1)/X-box binding protein 1 (XBP-1) branch of the UPR^ER^ regulates the activity of HSP-4. The endoribonuclease IRE-1 promotes the mRNA splicing of *xbp-1*. The spliced form of *xbp-1* (*xbp-1s*) encodes an active transcription factor that regulates *hsp-4* expression (16). Consistently, UV exposure increased the ratio of *xbp-1s* to unspliced *xbp-1* (*xbp-1u*) with or without DTT treatment (Fig. 2*C*). In addition, the expressions of several other XBP-1 target genes (17) were also induced by UV exposure (Fig. 2*C*), indicating the activation of the IRE-1/XBP-1 branch. Furthermore, *ire-1* RNAi could abolish UV-induced *hsp-4p*::GFP expression (Fig. 2*D*) and DTT resistance (Fig. 2*E*, Table S1), whereas RNAi targeting neither *atf-6* nor *pek-1*, genes of the other two branches of the UPR^ER^, had any effects on *hsp-4p*::GFP in response to UV (Fig. 2*D*). Together, these data suggest that DNA damage promotes ER stress resistance through the IRE-1/XBP-1 branch of the UPR^ER^.

### UV suppresses the apoptotic genes in somatic cells

**Figure 3:**
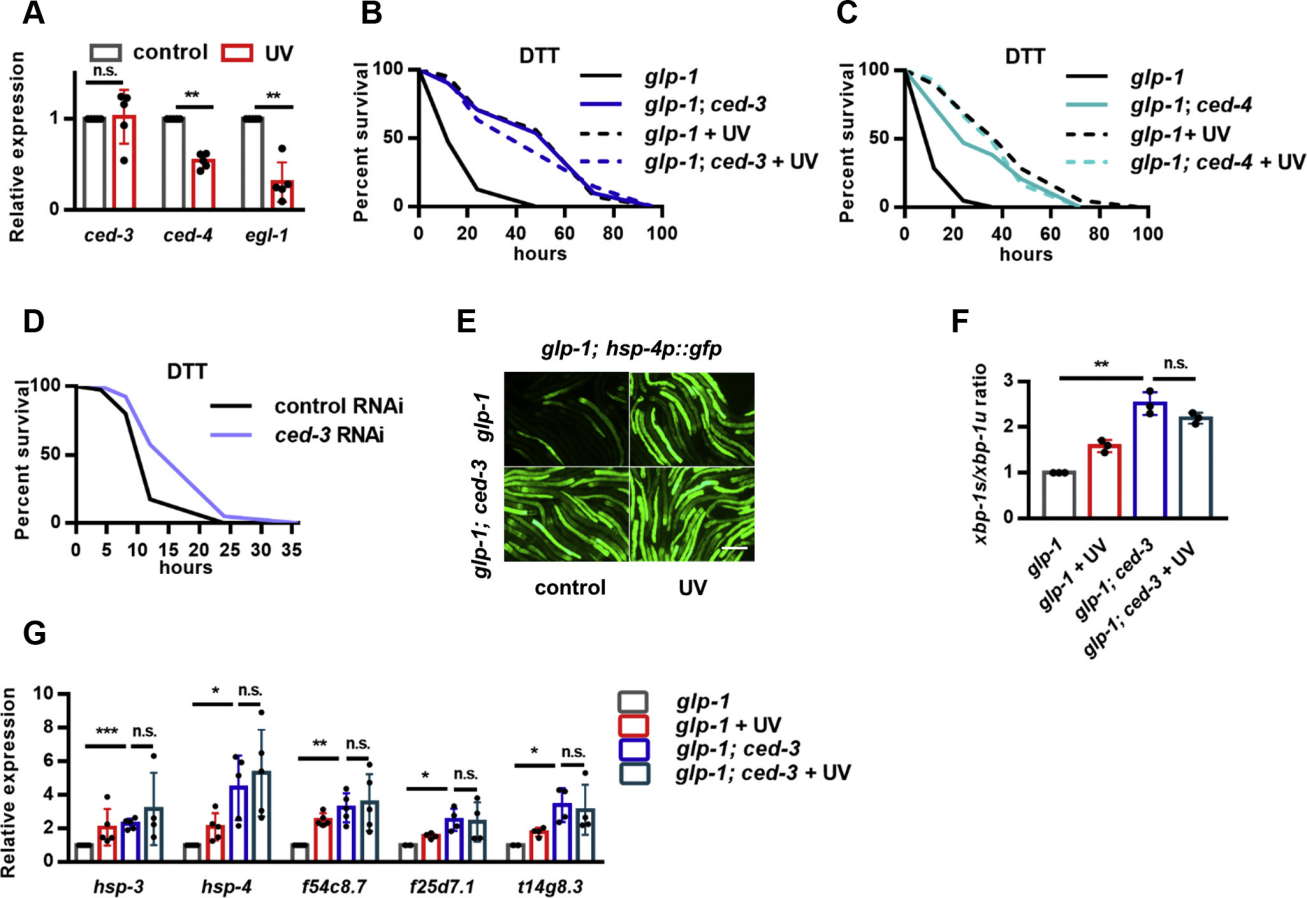
**Suppression of apoptotic genes promotes endoplasmic reticulum stress resistance in response to UV exposure.***A*, effects of UV on the expression of apoptotic genes in *glp-1* mutants. *B*–*C*, the mutation of *ced-3* (*B*) or *ced-4* (*C*) enhances endoplasmic reticulum stress resistance in *glp-1* mutants, and these effects cannot be enhanced by UV exposure. *D*, the intestine-specific RNAi of *ced-3* promotes the resistance to DTT in *glp-1* mutants. *E*–*G*, effects of *ced-3* mutation on the expression of *hsp-4p*::GFP (*E*), the ratio of *xbp-1s* to *xbp-1u* (*F*), and the mRNA levels of XBP-1 target genes (*G*) with or without UV treatment. ∗*p* < 0.05, ∗∗*p* < 0.01, ∗∗∗*p* < 0.001. The scale bar represents 100 μm. DTT, dithiothreitol; RNAi, RNA interference.

We mainly focused on DNA damage–induced ER stress responses in the following mechanistic study. A critical adaption to DNA damage in proliferating cells is apoptosis, which functions to prevent the spread of DNA damage. In *C. elegans*, apoptosis is initiated by the Bcl-2 homology 3 (BH3)-only protein egg laying defective-1 (EGL-1), which activates the cell death caspase cell death abnormality-3 (CED-3) by releasing CED-4 (18). Unexpectedly, we found that the expression of *ced-4* and *egl-1* was suppressed in germ line–deficient animals after UV exposure (Fig. 3*A*). Therefore, we next explored whether the suppression of the apoptotic pathway genes could elicit ER stress response. The results showed that the mutations of *ced-3* and *ced-4* could induce strong ER stress resistance in germ line–deficient animals (Fig. 3, *B*–*C*, Table S1). More importantly, *ced* mutations could not further enhance the ER stress resistance of UV-exposed worms (Fig. 3, *B*–*C*, Table S1), indicating that UV and *ced* genes act in a linear pathway. In addition, *ced-3* mutation could promote mitochondrial stress resistance but had no effects on the resistance to heat shock or oxidative stress (Fig. S3, *A–C*, Table S1), which again phenocopied the effects of UV exposure. We observed similar effects on ER stress resistance in *egl-1* knockdown worms (Fig. S3*D*, Table S1) but not in worms with mutation of *ced-13* (Fig. S3*E*, Table S1), the only other *C. elegans* gene encoding a BH3 domain–containing protein (19), suggesting that EGL-1, but not CED-13, is the BH3-only protein involved in UV-induced ER stress resistance.

A previous study reported that the mutations of apoptotic genes in the germ line could enhance the resistance to heat shock and ER stress in *C. elegans* (20), indicating different adaptive responses to *ced* mutations in the germ line and soma. To further confirm that the loss of function of *ced* in the soma is sufficient to promote ER stress resistance, we performed tissue-specific RNAi and found that *ced-3* knockdown specifically in the intestine could also increase ER stress resistance (Fig. 3*D*, Table S1).

As for the UPR^ER^ gene expression, *ced-3* mutants showed dramatically increased *hsp-4p*::GFP expression in response to ER stress, and this effect could not be further enhanced by UV exposure (Fig. 3*E*). In addition, mutation of *ced-3* could increase the ratio of *xbp-1s/xbp-1u* and the expression of other UPR^ER^ genes, which were not further enhanced in UV-exposed animals (Fig. 3, *F*–*G*). Collectively, these data support the idea that UV-induced DNA damage regulates ER stress response, at least partly, by suppressing the apoptotic genes.

### Unsaturated FAs are required for DNA damage–induced ER stress resistance

**Figure 4:**
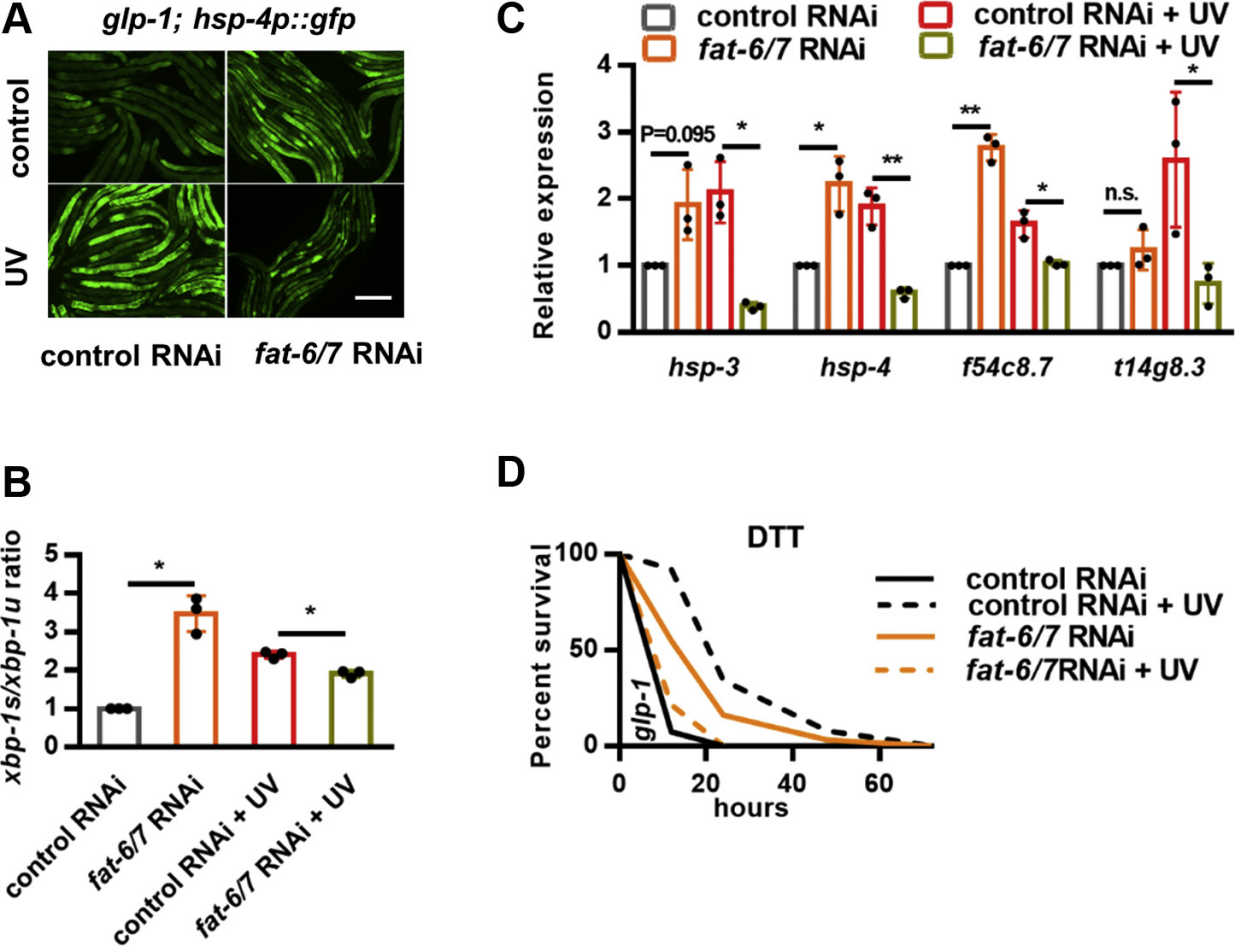
**DNA damage–induced endoplasmic reticulum (ER) stress resistance requires fatty acid desaturases FAT-6 and FAT-7.***A*–*B*, effects of *fat-6* and *fat-7* double RNAi on UV-induced *hsp-4p*::GFP expression (*A*), *xbp-1s/xbp-1u* ratio (*B*), ER unfolded protein response target gene expression (*C*), and ER stress resistance (*D*) in *glp-1* mutants. ∗*p* < 0.05, ∗∗*p* < 0.01. The scale bar represents 100 μm. DTT, dithiothreitol; RNAi, RNA interference.

ER is a central organelle not only for proteostasis but also for lipid biosynthesis. Major membrane lipids, such as PC and phosphatidylethanolamine (PE), are first synthesized in the ER and then transferred to other organelles. Moreover, lipid homeostasis in the ER has been functionally linked to the regulation of UPR^ER^ in multiple species (21, 22, 23, 24, 25). As such, we explored the relationship between lipid metabolism and DNA damage–induced ER stress resistance. We first evaluated FA composition by GC-MS/MS analysis and found that the contents of most FAs, mainly unsaturated ones, were significantly decreased after UV exposure (Fig. S4*A*), leading to a reduced ratio of unsaturated to saturated FAs (UFAs/SFAs) (Fig. S4*B*). Decreased FA unsaturation has been reported to promote the UPR^ER^ genes in *C. elegans* (25). *fat-6* and *fat-7* encode Δ9 desaturases that catalyze the first step of FA desaturation from stearic acid to oleic acid (OA) (Fig. S4*C*) (26, 27). We found that *fat-6/7* RNAi could indeed increase *hsp-4p*::GFP expression (Fig. 4*A*), which was suppressed by the addition of exogenous OA (Fig. S4*D*), confirming that the reduction of UFAs or UFA/SFA ratio could activate the UPR^ER^. Unexpectedly, we found that *fat-6/7* RNAi (which decreases UFAs), but not OA supplementation (which increases UFAs), completely abolished UV-induced *hsp-4p*::GFP expression (Fig. 4*A* and Fig. S4*E*), implying that in response to UV, the activation of the UPR^ER^ requires UFAs. Consistently, *fat-6/7* knockdown also suppressed the induction of the *xbp-1s/xbp-1u* ratio (Fig. 4*B*), expression of XBP-1 target genes (Fig. 4*C*), and ER stress resistance (Fig. 4*D*, Table S1) in response to UV. These findings suggest that UFAs are essential for the UPR^ER^ activation in UV-exposed somatic cells.

### Unsaturated PC is essential for UV-induced ER stress response

**Figure 5:**
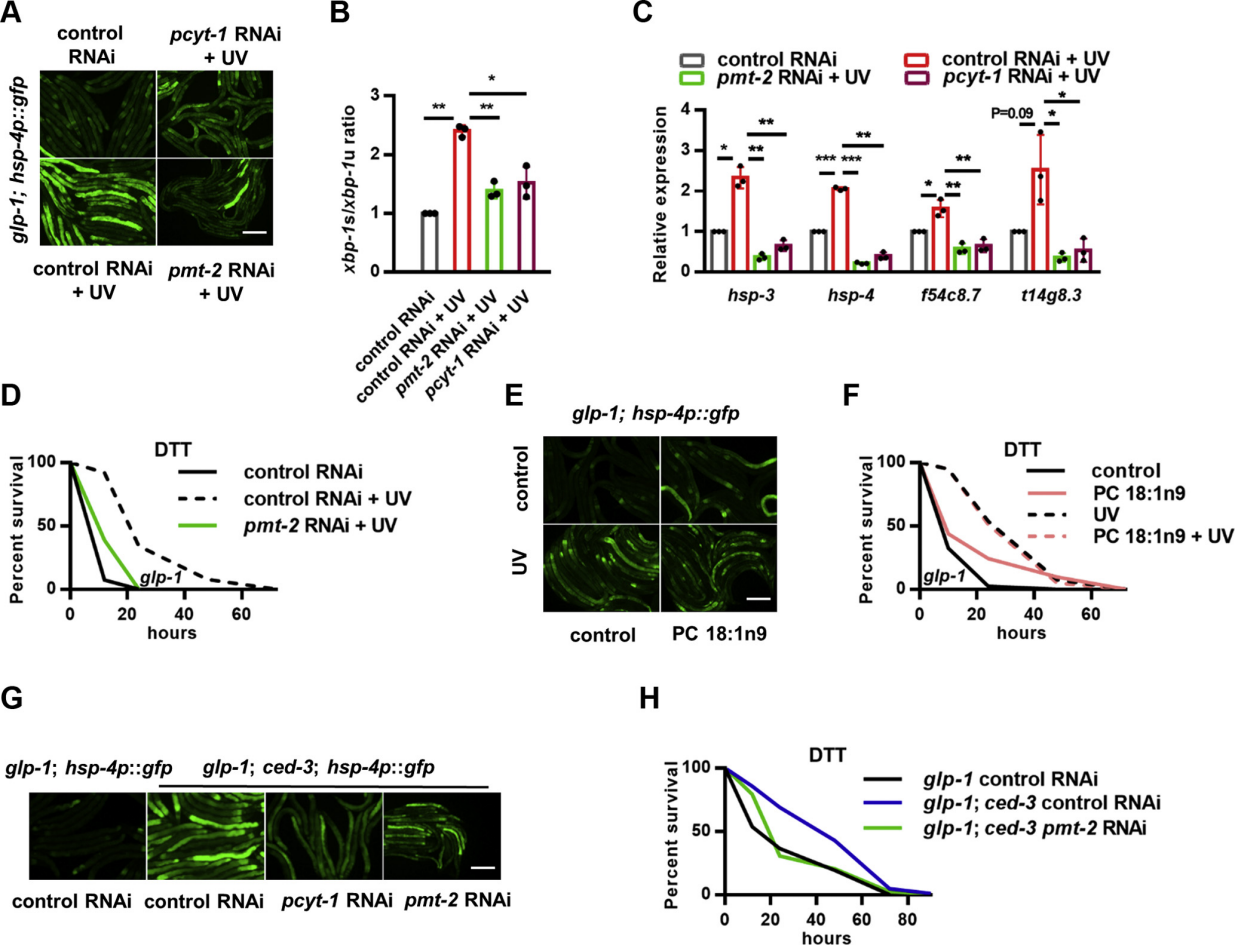
**DNA damage–induced endoplasmic reticulum (ER) stress resistance requires unsaturated PC.***A*–*C*, effects of *pmt-2* and *pcyt-1* RNAi on UV-induced *hsp-4p*::GFP expression (*A*), *xbp-1s/xbp-1u* ratio (*B*), and ER unfolded protein response target gene expression (*C*) in *glp-1* mutants. *D*, Effects of *pmt-2* RNAi on ER stress resistance in *glp-1* mutants exposed to UV. *E*–*F*, the effects of PC 18:1n9 supplementation on *hsp-4p*::GFP expression (*E*) and ER stress resistance (*F*) in *glp-1* mutants with or without UV exposure. (*G*) effects of *pcyt-1* and *pmt-2* RNAi on *ced-3* mutation–induced *hsp-4p*::GFP expression. (*H*) effects of *pmt-2* RNAi on ER stress resistance in germ line loss *ced-3* mutants. ∗*p* < 0.05, ∗∗*p* < 0.01, ∗∗∗*p* < 0.001. The scale bar represents 100 μm. DTT, dithiothreitol; PC, phosphatidylcholine; RNAi, RNA interference.

There seems to be a contradiction between the decreases of UFAs and the requirement of UFAs in UV-induced UPR^ER^ activation. Because FAs can serve as basic components of complex lipids and contribute to their physical and biological properties, we speculated that although the overall UFAs decreased after UV exposure (Fig. S4*A*), specific UFA-containing complex lipids might increase and function to enhance ER stress response. ER is a major compartment for PC synthesis and contains a large fraction of cellular PC; we therefore tested the involvement of PC in UV-induced response. In *C. elegans*, PC is synthesized from choline by the rate-limiting enzyme phosphocholine cytidylyltransferase-1 (Kennedy pathway) or generated by PMT-1/PMT-2–mediated sequential methylations from phosphoethanolamine (Fig. S5*A*) (28, 29). RNAi knockdown of either *pcyt-1* or *pmt-2* abrogated UV-induced *hsp-4p*::GFP expression (Fig. 5*A*), *xbp-1s/xbp-1u* ratio (Fig. 5*B*), and UPR^ER^ genes expression (Fig. 5*C*), implying that PC production is required for the activation of the IRE-1/XBP-1 during DNA damage. Accordingly, *pmt-2* RNAi also suppressed ER stress resistance (Fig. 5*D*, Table S1). Furthermore, we validated that *pmt-2* RNAi could indeed decrease most unsaturated PC (Fig. S5, *B*–*C*), whereas the contents of saturated PC were elevated (Fig. S5, *B*–*C*), which may be due to compensatory responses for PC maintenance. These data are consistent with the essential roles for UFAs in adaptive response to UV and suggest that UFAs may function in the form of unsaturated PC to induce ER stress resistance.

We next examined whether the supplementation of specific PC could promote the UPR^ER^ by feeding worms with PC containing different FA chains. Intriguingly, OA-containing PC (PC 18:1n9), but not saturated PC (PC 16:0, PC 18:0), significantly increased *hsp-4p*::GFP expression (Fig. 5*E* and Fig. S5*D*) and ER stress resistance (Fig. 5*F* and Fig. S5*E*, Table S1). Furthermore, PC 18:1n9 and UV showed no additive effects (Fig. 5, *E*–*F*, Table S1), suggesting that they act in a linear pathway. In addition, PC 18:1n9 supplementation could partially reverse the effects of *pmt-2* RNAi on ER stress resistance (Fig. S5*F*, Table S1). These results suggest PC, probably unsaturated PC, plays crucial roles in UV-induced UPR^ER^ activation in the soma of *C. elegans*.

We further examined whether PC acts downstream of *ced* genes in the regulation of ER stress responses. As expected, inhibition of unsaturated PC synthesis abrogated the induction of *hsp-4p*::GFP (Fig. 5*G*) and ER stress resistance (Fig. 5*H*) in *ced-3* mutants, supporting a UV-CED-PC pathway in regulating ER stress responses in *C. elegans*.

**Figure 6:**
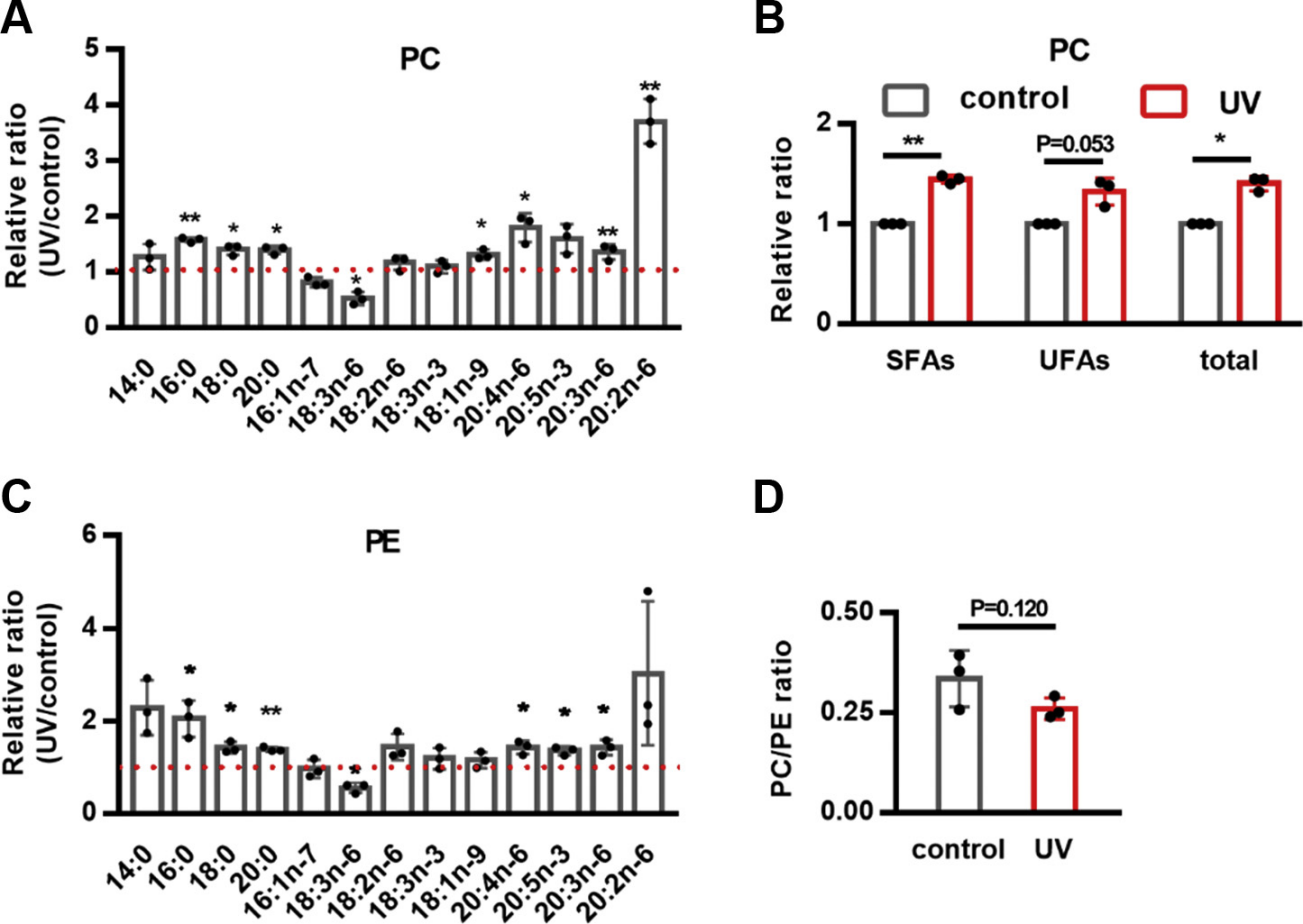
**DNA damage regulates PC contents.***A*, effects of UV on the individual fatty acid chain of PC in *glp-1* mutants. *B*, UV exposure increases the SFA chains, UFA chains, and total FA chains of PC in *glp-1* mutants. *C*, effects of UV on the individual fatty acid chain of PE in *glp-1* mutants. *D*, Effects of UV on the PC/PE ratio in *glp-1* mutants. ∗*p* < 0.05, ∗∗*p* < 0.01. PC, phosphatidylcholine; PE, phosphatidylethanolamine; SFAs, saturated fatty acids; UFAs, unsaturated fatty acids.

Next, we examined how UV regulated PC composition by examining their acyl chains. Intriguingly, although most UFAs were reduced after UV exposure (Fig. S4*A*), the contents of unsaturated PC as well as saturated PC were elevated (Fig. 6, *A*–*B*), resulting in an increase of total cellular PC (Fig. 6*B*). In addition, UV increased the contents of several PE species (Fig. 6*C*), whereas the ratio of PC/PE was not significantly affected (Fig. 6*D*). These data suggest that UV may promote the production of several complex lipids, among which unsaturated PC is required to activate the UPR^ER^.

### SKN-1 negatively regulates UV-induced ER stress resistance

**Figure 7:**
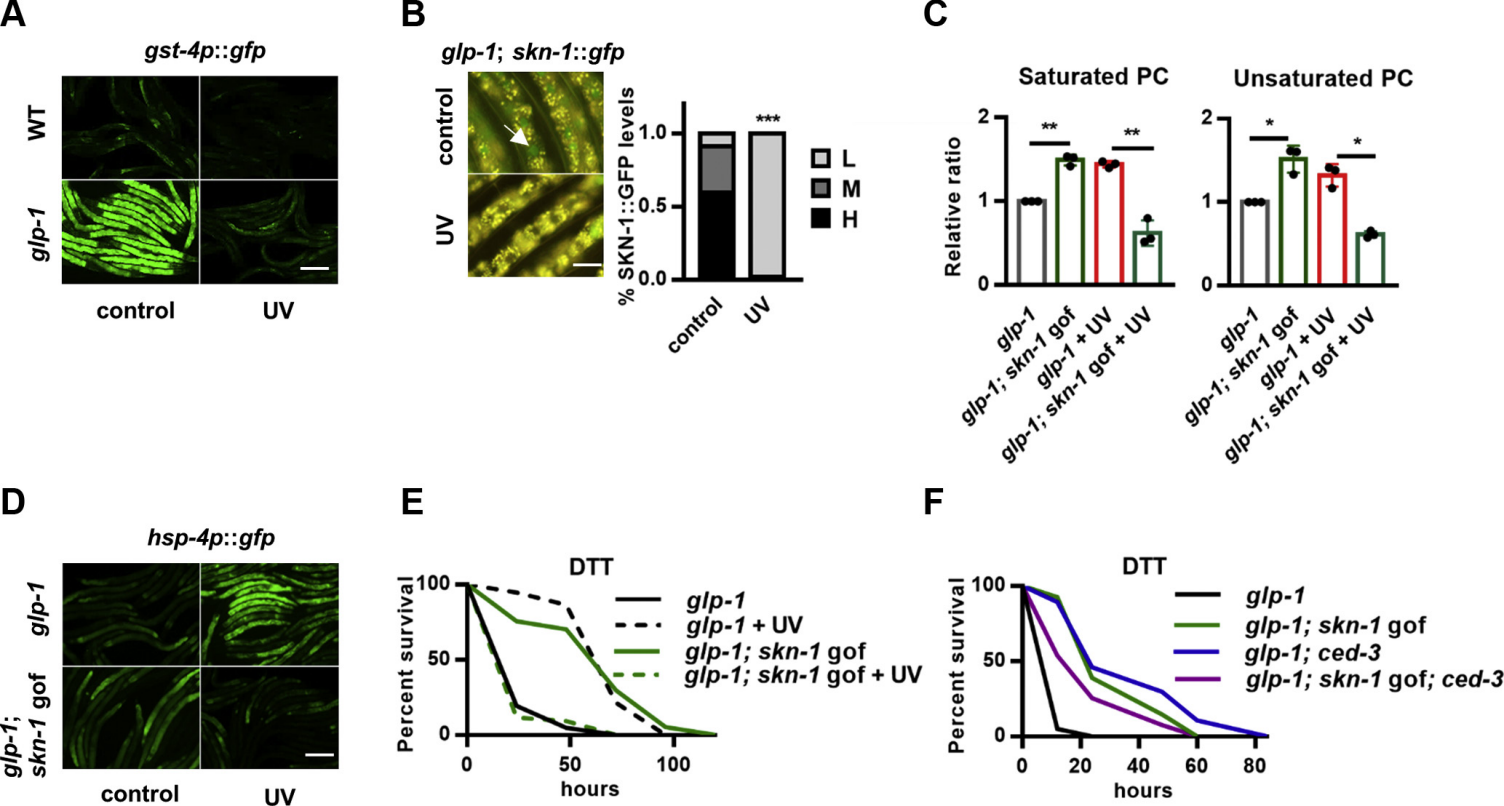
**SKN-1 suppression ensures PC increases and endoplasmic reticulum stress resistance in response to DNA damage.***A*, UV suppresses the expression of *gst-4p*::GFP in WT and *glp-1* mutants. *B*, the nuclear occupancy of SKN-1::GFP in *glp-1* mutants in response to UV exposure. Left panel, representative images; right panel: quantification data. The *arrow* indicates the nuclear GFP signal. The nuclear occupancy of SKN-1::GFP was scored as low (L), medium (M), and high (H). *C*, eEffects of the *skn-1* gain-of-function (gof) mutation on PC contents in response to UV in *glp-1* mutants. *D*–*E*, the *skn-1* gof mutation abolishes UV-induced *hsp-4p*::GFP expression (*D*) and endoplasmic reticulum stress resistance (*E*) in *glp-1* mutants. *F*, effects of the *skn-1* gof mutation on DTT resistance in germ line–deficient *ced-3* mutants. ∗*p* < 0.05, ∗∗*p* < 0.01, ∗∗∗*p* < 0.001. The scale bar represents 100 μm for panels *A* and *D* and 20 μm for panel *B*. DTT, dithiothreitol; PC, phosphatidylcholine.

The increase of total cellular PC in UV-exposed worms requires the incorporation of FAs as their acyl chains, which implies that FA metabolism is also regulated during UV adaptation. SKN-1 is one of the core transcription factors that controls FA metabolism in *C. elegans*. The *skn-1* loss-of-function mutation increases fat contents, whereas the *skn-1* gain-of-function (*skn-1* gof) mutation suppresses lipid accumulation (30, 31, 32). We therefore evaluated whether SKN-1 was regulated on UV exposure and found that the basal activity of the SKN-1 reporter *gst-4p*::GFP was suppressed (Fig. 7*A*), whereas the *skn-1* gof mutation resisted the suppression (Fig. S6*A*). Similarly, the mRNA expression of *gst*-*7*, another SKN-1 target, was also reduced on UV exposure (Fig. S6*B*). As a transcription factor, SKN-1 translocates into the nucleus once activated (33), and we did observe an apparent nuclear exclusion of SKN-1::GFP in germ line–deficient *C. elegans* after UV exposure (Fig. 7*B*). Collectively, SKN-1 activity was suppressed in *C. elegans* on UV exposure.

SKN-1 functions to reduce FA contents (30, 32, 34) (Fig. S6*C*); we therefore speculated that UV suppressed SKN-1 to ensure PC production, which is required for subsequent UPR^ER^ activation. To test the hypothesis, we first examined the *skn-1* gof mutants that could resist UV-induced SKN-1 inhibition (Fig. S6*A*) and found that UV-induced increases of PC and PE, including saturated and unsaturated ones, were dramatically compromised by the *skn-1* gof mutation (Fig. 7*C* and Fig. S6*D*). These data support that when SKN-1 is not suppressed, *C. elegans* somatic cells fail to increase unsaturated PC in response to DNA damage. We also noticed that the *skn-1* gof mutation could increase PC contents in the absence of UV exposure (Fig. 7*C*), suggesting that SKN-1 regulates PC in a context-dependent manner.

Considering the critical roles for unsaturated PC in UV-induced UPR^ER^ responses, SKN-1 activation might negatively affect UV-induced stress resistance. SKN-1 has been reported to activate the UPR^ER^ gene *hsp-4* (35), and we did observe a basal induction of *hsp-4p*::GFP and ER stress resistance in the *skn-1* gof mutants (Fig. 7, *D*–*E*, Table S1), which was consistent with the unsaturated PC contents in the absence of UV (Fig. 7*C*). However, when exposed to UV, the *skn-1* gof mutation dramatically abrogated the activation of *hsp-4p*::GFP (Fig. 7*D*) and ER stress resistance (Fig. 7*E*, Table S1). In contrast, the *skn-1* loss-of-function mutation had no apparent effects on UV-induced ER stress resistance (Fig. S6*E*, Table S1). Moreover, the *skn-1* gof mutation suppressed ER stress resistance in *ced-3* mutants (Fig. 7*F*, Table S1) and in animals treated with *exo-3* or *apn-1* RNAi (Fig. S6*F*, Table S1). These data collectively suggest that SKN-1 activation counteracts DNA damage–induced UPR^ER^ activation, and SKN-1 suppression is required to increase unsaturated PC and to promote UPR^ER^ activation in response to DNA damage.

## Discussion

The present study uncovered how *C. elegans* adapt to DNA damage. We demonstrated that cells suppress the apoptotic genes to elicit stress responses, which may promote cellular preservation. The *C. elegans* soma expresses low levels of classic DNA damage response genes, such as the checkpoint genes and several DNA repair genes (8, 36), suggesting that in response to DNA damage, the somatic cells evolve to boost cell maintenance rather than target the damages. Consistent with this idea, *C. elegans* carrying the checkpoint gene mutation are thermotolerant and long lived (37), and nematodes with DNA mismatch repair defects are oxidative stress resistant (38). Together, these studies and our data strongly support tight and complex links between the DNA damage response and the stress response pathways.

It should be noted that cell nonautonomous effects could also impact the cellular response to DNA damage in *C. elegans*. DNA damage in germ cells confers somatic stress resistance, which prolongs the somatic endurance to allow the delayed offspring generation when germ cells are genomically compromised (13). Considering the importance of the supportive soma to germ cells, the cell-autonomous adaptation to DNA damage in the soma may not only be critical for somatic cell maintenance but also be beneficial for reproduction.

The somatic cells of *C. elegans* are postmitotic, raising an interesting possibility that the observed UPR^ER^ response is specific for postmitotic cells. Postmitotic cells respond to DNA damage in a way different from that in proliferating cells, likely because of their irreplaceable nature. Maintenance for survival and function in these cells is extremely crucial for tissue homeostasis, and generally these cells are highly resistant to cell death (39, 40). Consistently, we showed that UV exposure suppresses apoptotic genes and promotes stress resistance in the soma of *C. elegans*. In contrast, the UV dosage used in the present study has been reported to cause acute apoptosis in the germ line of *C. elegans* (41). In addition, our work indicates activation of the IRE-1/XBP-1 branch of the UPR^ER^ on DNA damage as a cellular maintenance program, whereas recent works suggest it might not be the case in proliferating cells. A study of mouse embryonic fibroblasts revealed that DNA damage engages regulated IRE1α-dependent decay to modulate DNA damage response genes, without activating XBP1 and canonical UPR genes (42). Moreover, DNA damage in cancer cell line HCT116 even suppresses IRE1α-XBP1 to stabilize proapoptotic protein BCL-2 interacting killer that contributes to apoptotic cell death (43), suggesting that DNA damage appears not to activate canonical UPR^ER^ in proliferating cells. The specificity or general application of current observation in other types of cells deserves further studies.

The present study revealed an unexpected suppression of apoptotic genes by UV exposure in *C. elegans* somatic cells, which is consistent with a previous report that the soma of adult *C. elegans* is typically believed not to undergo apoptosis even in response to DNA damage (7). More intriguingly, the stimulation of the apoptotic pathway in the *C. elegans* soma was found to activate a protective program but not apoptosis (44). Therefore, the function of apoptotic genes on somatic stress responses may be independent of apoptosis, which needs further investigation.

Our data showed that UV exposure reduces the contents of multiple FAs, the products of *de novo* lipogenesis, which is consistent with a previous report that DNA damage in *C. elegans* could reduce the protein levels of enzymes involved in *de novo* lipogenesis (6). Intriguingly, ER stress has been reported to promote fat accumulation by activating sterol regulatory element-binding proteins (SREBPs) that are known to drive lipid biosynthetic genes (45, 46, 47, 48, 49). Furthermore, several caspases that are activated during apoptosis were found to induce SREBP processing (46, 50, 51). Because we observed reduced expression of apoptotic genes in postmitotic cells during DNA damage, it is possible that the enhanced ER stress response may suppress *de novo* lipogenesis in a way that involves SREBPs and caspases. How DNA damage regulates lipogenesis and how this metabolic adaptation affects DNA damage response deserve further exploration.

Previous studies have uncovered that the UPR^ER^ can be activated in response to the lipid changes of the ER, named lipid bilayer stress. For example, when the ER contents of PC are reduced, the conserved core UPR^ER^ component IRE1 can directly sense the decrease and initiate the subsequent UPR response (23). However, in our study, the UV-induced UPR^ER^ actually requires the participation of unsaturated PC, implying that mechanisms different from lipid bilayer stress may be involved. Indeed, specific PC species have been reported to directly bind and regulate cellular signaling proteins, such as mammalian peroxisome proliferator activated receptor alpha (52) and *C. elegans* nuclear hormone receptor family-25 (53). In addition, PC was found to be required for maintaining the stabilities of some membrane-bound proteins located on the ER and mitochondria (54, 55). The precise mechanism of how unsaturated PC activates the IRE-1XBP-1 branch of the UPR^ER^ deserves further exploration.

In summary, we report the mechanism of how *C. elegans* somatic cells adapt to DNA damage to maintain cellular homeostasis and to favor organismal survival, which may be an ancestral adaptation shared by mammalian cells.

## Experimental procedures

### *C. elegans* strains and maintenance

*C. elegans* were cultured on standard nematode growth medium (NGM) seeded with *Escherichia coli* OP50-1 (56). The following strains were provided by *Caenorhabditis* Genome Center: wild type N2 Bristol, CB4037[*glp-1*(*e2141*)], MHG171[*sid-1*(*qt9*); *alxIs9*(*vha-6p::sid-1::SL2::GFP*)], SJ4005[*hsp-4p::gfp*], SJ4100[*hsp-6p::gfp*], MT1522[*ced-3*(*n717*)], MT2547[*ced-4*(*n1162*)], FX536[*ced-13*(*tm536*)], SPC207[*skn-1*(*lax120*)], VC1772[*skn-1*(*ok2315*)*/nT1*], LD1[*skn-1b/c::gfp*], and CL2166[*gst-4p*::*gfp*]. Double and triple mutants were generated by using standard genetic methods.

### Stress resistance assays

Day 1 adult *glp-1* worms were exposed to UV-C radiation of 400 J/m^2^, and the resistances to multiple stresses were measured at day 3. For heat shock resistance assay, worms were cultured at 35 °C for survival analysis. For tert-butyl hydroperoxide (Sigma) resistance, worms were transferred to NGM plates supplemented with 10 mM of tert-butyl hydroperoxide for survival analysis. For DTT resistance, worms were transferred to NGM plates supplemented with 7.5 mM DTT for survival analysis. For malonic acid (Sigma) resistance, worms were transferred to NGM plates supplemented with 45 mM of malonic acid for survival analysis.

### Fluorescent microscopy

For fluorescent analysis, worms were exposed to UV at day 1 adult stage and 24 h later treated with the following drugs to induce GFP expression: 3.75 mM of DTT or 9 μg of tunicamycin for *hsp-4p*::GFP and 5 μg of antimycin for *hsp-6p*::GFP. Then microscopic imaging was performed as previously described (57). Briefly, worms were paralyzed with 1 mM of levamisole, and fluorescent microscopic images were taken after mounted on slides. To study the SKN-1 nuclear localization, SKN-1::GFP worms were mounted on slides. The levels of GFP nuclear localization were scored. Briefly, no nuclear GFPs, GFP signal in the nucleus of anterior or posterior intestine cells and nuclear GFP in all intestinal cells, are categorized as of low, medium, and high expression, respectively.

### qRT-PCR

Quantitative RT-PCR was performed as previously described (57, 58). Briefly, worms were collected, washed in M9 buffer, and then homogenized in Trizol reagent (Life Technologies). RNA was extracted according to the manufacturer's protocol. DNA contamination was digested with DNase I (Thermo Fisher Scientific), and subsequently, RNA was reverse transcribed to complementary DNA by using the RevertAid First Strand cDNA synthesis Kit (Thermo Fisher Scientific). Quantitative PCR was performed using SYBR Green (Bio-Rad). The expression of *snb-1* was used to normalize samples.

### RNA interference treatment

HT115 bacteria containing specific dsRNA-expression plasmids (Ahringer library) (59) were cultured overnight at 37 °C in LB containing 100 μg/ml of carbenicillin and seeded onto NGM plates containing 5 mM of isopropyl b-D-1-thiogalactopyranoside. RNAi was induced at 25 °C for 24 h. L1 worms were added to those plates to knockdown indicated genes. For intestine-specific RNAi, strain MHG171[*sid-1(qt9)*; *alxIs9(vha-6p::sid-1::SL2::GFP)*] was used (60).

### FA quantification

FA contents were measured as previously described (26) with some modifications. About 500 to 1000 age-synchronized adult worms were washed off plates and washed three times with water. Worm pellets were resuspended with 1.2 ml of 2.5% sulfuric acid in methanol and incubated at 80 °C for 1 h. Then, 1 ml of supernatant was mixed with 1.2 ml of hexane and 1.8 ml of water to extract FA methyl esters (FAMEs) for GC-MS analysis. The Supelco 37 Component FAME Mix (Sigma) was used to determine the retention time. The Shimadzu GCMS-TQ8040 Gas Chromatograph Mass Spectrometer equipped with SH-Rxi-5sil MS column was used.

### Quantifications of PC and PE

TLC was performed as previously described (26) with modifications. About 50,000 L4 worms were collected and washed with M9 to remove bacteria and were sonicated in 0.25 ml of PBS. Then 5-ml mixture of ice-cold chloroform: methanol (1:1) was added and mixed immediately and incubated overnight at −20 °C with occasional shaking to extract lipids. Then, 2.2 ml of Hajra's solution (0.2 M of phosphoric acid, 1 M of potassium chloride) was added, and the lower organic phase containing the lipids was recovered by centrifuging for 1 min at 3000 rpm and dried under nitrogen. Dried lipids were resuspended in chloroform for TLC separation.

The silica gel TLC plate was activated by incubating at 110 °C for 75 min. The samples were loaded onto the TLC plate along with lipid standards. The plate was run with a chloroform:methanol:water:acetic acid solvent mixture (65:43:3:2.5) until the solvent front was three-fourths of the way up the plate. The plate was dried and run with a new solvent mixture of hexane:diethyl ether:acetic acid (80:20:2) until the solvent front reached the top of the plate. Lipids were visualized under UV light after spraying the plate with 0.005% of primuline, and spots corresponding to the major phospholipids were scraped to tube, resuspended in 2.5% of sulfuric acid in methanol, and incubated for 1 h at 80 °C to create FAMEs for GC-MS/MS analysis. About 50 μg of tridencanoic acid was added to each tube as an internal standard.

### Lipid supplementation

OA was dissolved in dimethyl sulfoxide to make a 300 mM of stock solution, and 7.5 μl of each was added onto the surface of NGM plates. For PC supplementation, PC species were dissolved in dimethyl sulfoxide to make 17 mM of stock solutions, and 7.5 μl of each was added onto the surface of NGM plates.

### Quantification and statistical analysis

Data were presented as mean ± S.D. Survival data were analyzed by using log-rank (Mantel–Cox) tests. The levels of fluorescent micrographs were analyzed by using Chi-squared and Fisher exact tests. The quantitative PCR and GC data were analyzed by using paired Student *t* tests. A *p* value of < 0.05 was considered as significant.

## Data availability

All data are contained within the article.

## Conflict of interest

The authors declare that they have no conflicts of interest with the contents of this article.
