# Supplementary material for: DNA damage promotes ER stress resistance through elevation of unsaturated phosphatidylcholine in Caenorhabditis elegans
Source: J Biol Chem. 2020 Nov 24;296:100095. doi: 10.1074/jbc.RA120.016083 (PMC7949029; doi:10.1074/jbc.RA120.016083)
Supplement: Supplementary file 1 — Figures S1–S6 and Table S1 [file mmc1.pdf]

DNA damage promotes ER stress resistance through elevation of unsaturated phosphatidylcholine in *C. elegans*

Jianhui Deng[#], Xue Bai[#], Haiqing Tang* and Shanshan Pang*

School of Life Sciences, Chongqing University, Chongqing, 401331, China

[#]Co-first authors

*Corresponding authors: Haiqing Tang, Shanshan Pang

E-mail: hqtang@cqu.edu.cn, sspang@cqu.edu.cn

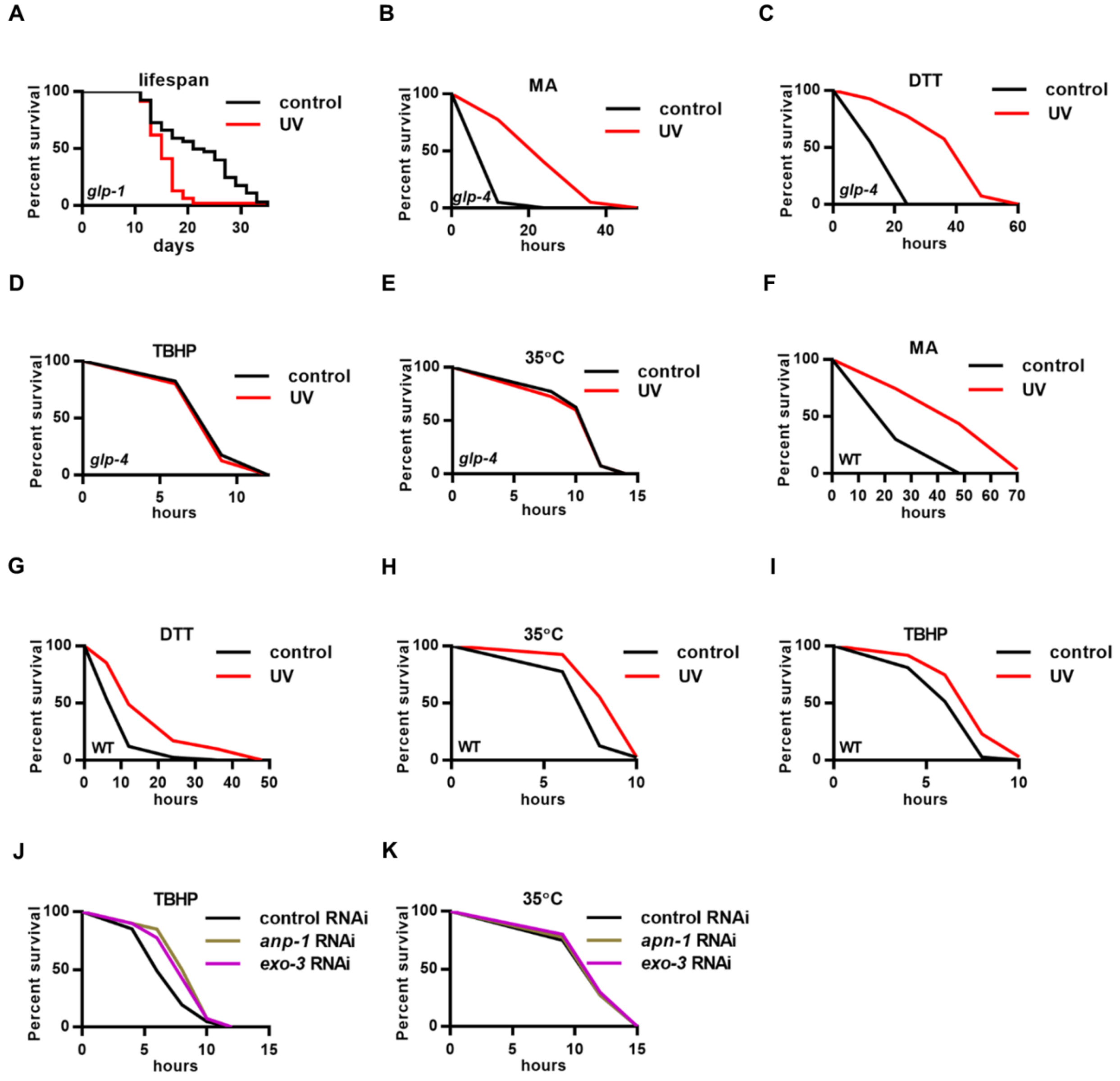

Figure S1. DNA damage regulates stress resistance in the somatic cells of *C. elegans*. (A) Day1 adult *glp-1* mutants were exposed to UV-C radiation of 400 J/m2 and the lifespan was measured thereafter. (B-E) Survival of the UV-exposed *glp-4* mutants in response to MA (B), DTT (C), heat stress (D) and TBHP (E). (F-I) Survival of the UV-exposed wild-type (WT) animals in response to MA (F), DTT (G), TBHP (H) and heat stress (I). (J-K) The intestine-specific RNAi of *apn-1* or *exo-3* (by using an intestine-specific RNAi strain) promotes the resistance to TBHP (J) but not to 35 ℃ heat shock (K) in germline-deficient *C. elegans*.

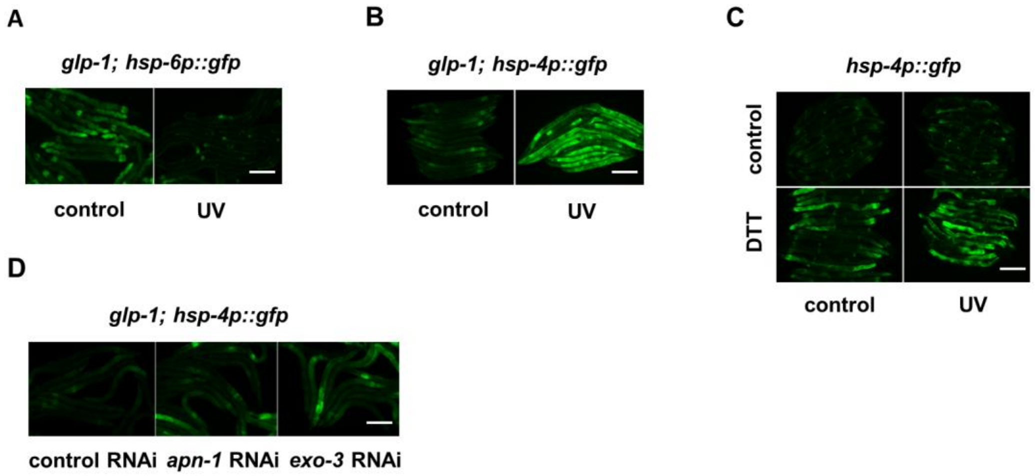

Figure S2. DNA damage induces the UPR^{ER} reporter. (A) UV exposure suppresses *hsp-6p*::GFP expression induced by antimycin in *glp-1* mutants. (B) UV exposure elevates the expression of *hsp-4p*::GFP in *glp-1* mutants in response to tunicamycin. (C) UV-exposed wild-type animals show elevated expression of *hsp-4p*::GFP in response to DTT. (D) The RNAi of *apn-1* or *exo-3* elevates the expression of *hsp-4p*::GFP induced by DTT in *glp-1* mutants. Scale bar = 100µm.

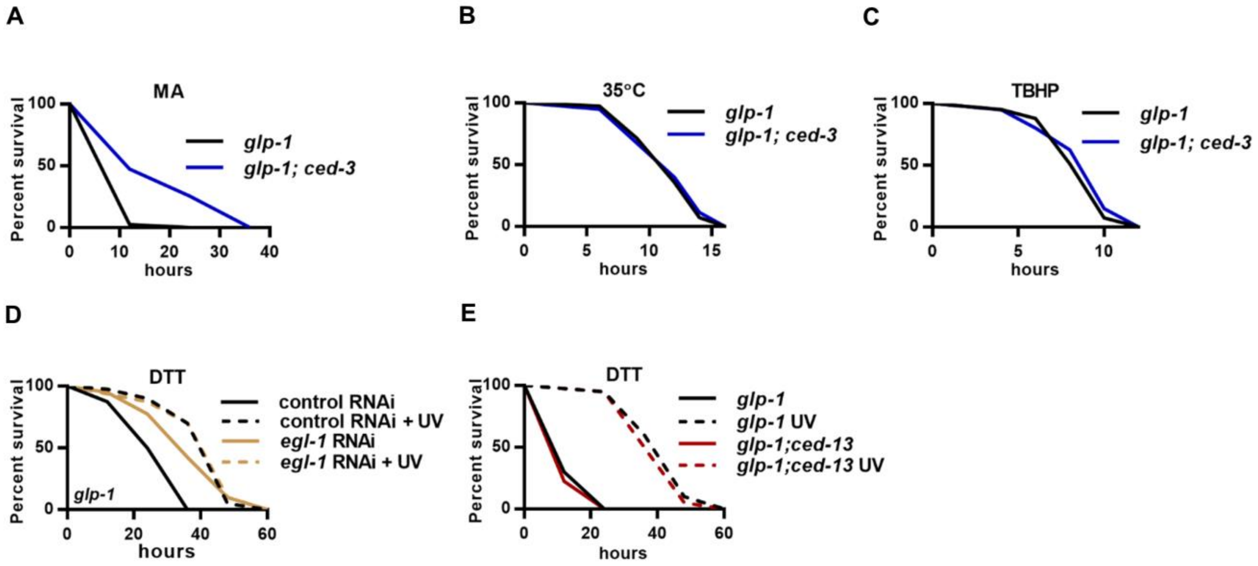

Figure S3. Apoptotic pathway genes affect stress resistance in germline-deficient animals. (A-C) Effects of *ced-3* mutation on animal resistance to MA (A), 35 ℃ heat shock (B) and TBHP (C) in *glp-1* mutants. (E-F) Effects of *egl-1* RNAi (E) and *ced-13* mutation (F) on DTT resistance in *glp-1* mutants with or without UV exposure.

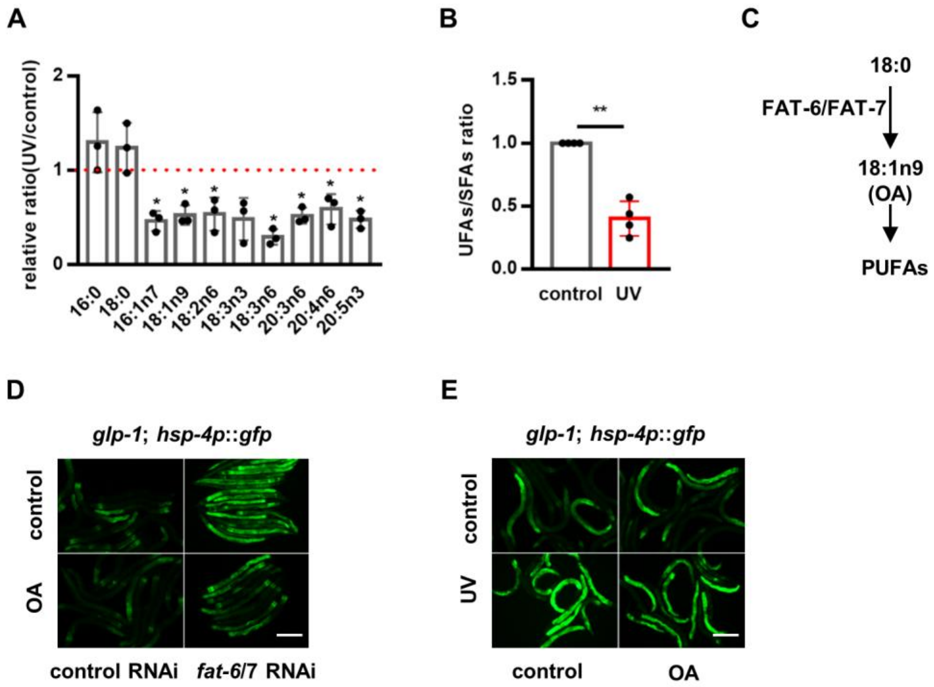

Figure S4. DNA damage-induced ER stress resistance requires unsaturated fatty acids. (A) Effects of UV exposure on the contents of major fatty acid species in *glp-1* mutants. (B) UV exposure decreases the ratio of UFAs to SFAs in *glp-1* mutants. (C) The schematic of fatty acid desaturation in *C. elegans*. (D-E) Effects of OA addition on *fat-6/7* RNAi-induced (C) and UV-induced (D) *hsp-4p*::GFP expression in *glp-1* mutants. * $P < 0.05$, ** $P < 0.01$. Scale bar = 100μm.

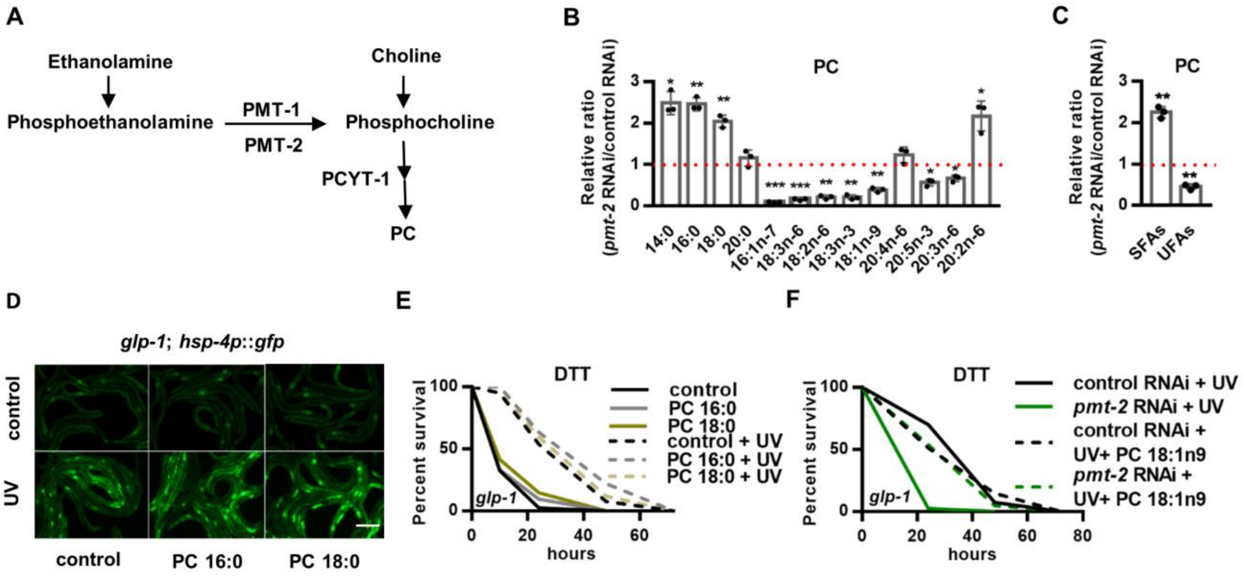

Figure S5. DNA damage-induced ER stress resistance requires unsaturated PC. (A) The schematic of PC synthesis pathways in *C. elegans*. (B) Effects of *pmt-2* RNAi on individual fatty acid chain of PC. (C) Effects of *pmt-2* RNAi on the SFA and UFA chains of PC. (D-E) Effects of saturated PC on *hsp-4p*::GFP expression (D) and ER stress resistance (E) in *glp-1* mutants with or without UV exposure. (F) PC 18:1n9 supplementation reverses the effects of *pmt-2* RNAi on DTT resistance in UV-exposed *glp-1* mutants. * P < 0.05, ** P < 0.01, *** P < 0.001. Scale bar = 100μm.

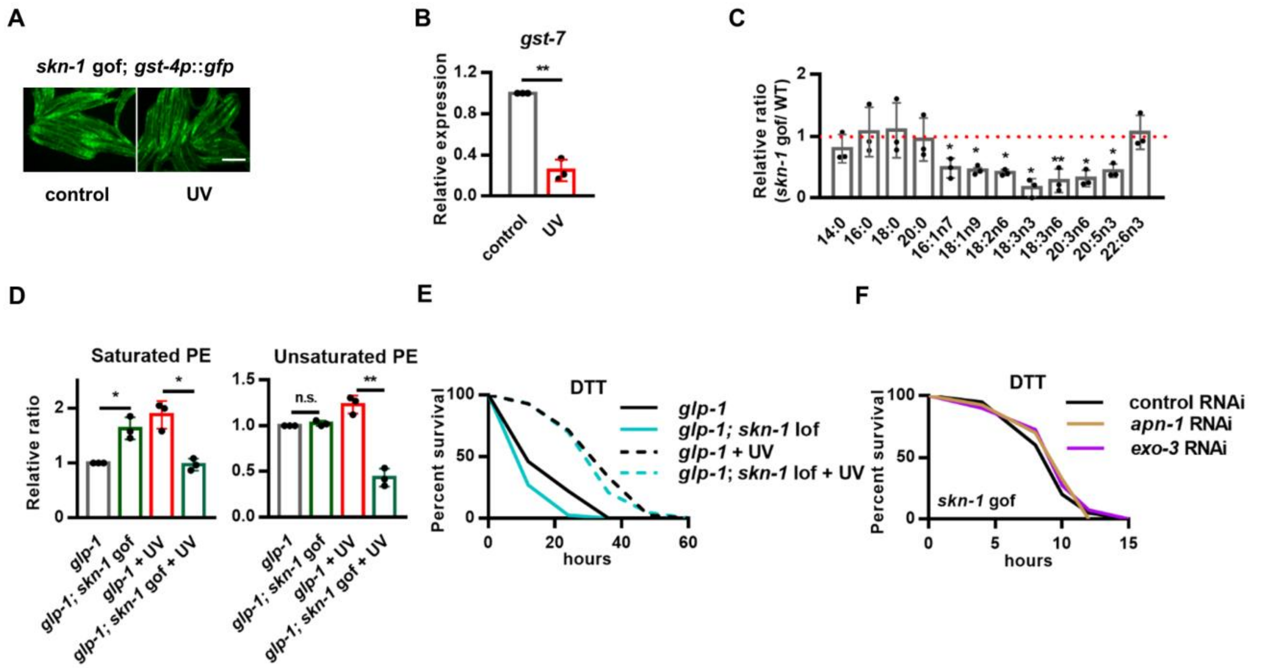

Figure S6. UV suppresses SKN-1 to ensure the induction of ER stress resistance. (A) Effects of the *skn-1* gof mutation on the expression of *gst-4p*::GFP in response to UV exposure. (B) UV exposure suppresses the mRNA expression of *gst-7* in *glp-1* mutants. (C) Effects of the *skn-1* gof mutation on the contents of major fatty acid species. (D) Effects of the *skn-1* gof mutation on PE contents in response to UV exposure in *glp-1* mutants. (E) The *skn-1* lof mutation has no effects on UV-induced ER stress resistance. (F) The intestine-specific knockdowns of the BER genes have no effects on ER stress resistance in *glp-1* mutants with the *skn-1* gof mutation. * $P < 0.05$, ** $P < 0.01$. Scale bar = 100μm.

Table S1. Survival data. Repeats 1 were graphed in figures.

Figures	Strain/Treatment	Mean survival time ± SEM (hours)	# Worms Censored/Total	P value
**1A**	*glp-1*	10.68 ± 0.42	2/36	
**Repeat 1**	*glp-1* UV	10.30 ± 0.48	0/37	0.654[a]
**1A**	*glp-1*	12.08 ± 0.42	0/36	
**Repeat 2**	*glp-1* UV	12.08 ± 0.37	0/39	0.8453[a]
**1A**	*glp-1*	11.85 ± 0.41	0/40	
**Repeat 3**	*glp-1* UV	12.08 ± 0.42	0/40	0.6412[a]
**1B**	*glp-1*	8.84 ± 0.28	0/43	
**Repeat 1**	*glp-1* UV	8.71 ± 0.29	0/42	0.857[a]
**1B**	*glp-1*	11.95 ± 0.30	0/40	
**Repeat 2**	*glp-1* UV	11.67 ± 0.29	0/45	0.3899[a]
**1B**	*glp-1*	12.00 ± 0.29	0/42	
**Repeat 3**	*glp-1* UV	11.92 ± 0.47	0/39	0.5882[a]
**1C**	*glp-1*	12.29 ± 0.29	2/36	
**Repeat 1**	*glp-1* UV	60.58 ± 3.00	0/37	<0.001[a]
**1C**	*glp-1*	12.36 ± 0.36	0/33	
**Repeat 2**	*glp-1* UV	43.31 ± 3.80	0/34	<0.001[a]
**1C**	*glp-1*	13.37 ± 0.94	0/35	
**Repeat 3**	*glp-1* UV	49.37 ± 3.06	0/35	<0.001[a]
**1D**	*glp-1*	29.85 ± 1.99	0/41	
**Repeat 1**	*glp-1* UV	73.53 ± 2.47	3/40	<0.001[a]
**1D**	*glp-1*	17.86 ± 1.33	0/43	
**Repeat 2**	*glp-1* UV	34.77 ± 1.89	0/39	<0.001[a]
**1D**	*glp-1*	15.80 ± 1.05	0/41	
**Repeat 3**	*glp-1* UV	41.85 ± 1.50	0/41	<0.001[a]
**1E**	*glp-1* control	13.80 ± 0.68	0/40	
**Repeat 1**	*glp-1* 4 J/m^2	18.51 ± 1.22	0/35	0.0012[a]
	glp-1 40 J/m^2	21.60 ± 1.35	0/35	<0.001[a]
	glp-1 400 J/m^2	33.90 ± 1.03	0/40	<0.001[a]
**1E**	*glp-1* control	15.08 ± 0.95	0/39	

**Repeat 2**	*glp-1* 4 J/m^2	19.50 ± 1.10	0/40	0.0042 [a]
	glp-1 40 J/m^2	19.58 ± 1.37	0/38	0.0097[a]
	glp-1 400 J/m^2	23.40 ± 0.73	0/40	<0.001[a]
**1E**	*glp-1* control	13.80 ± 0.68	0/40	
**Repeat 3**	*glp-1* 4 J/m^2	22.80 ± 1.46	0/40	<0.001[a]
	glp-1 40 J/m^2	22.20 ± 1.97	1/40	<0.001[a]
	glp-1 400 J/m^2	29.70 ± 1.90	0/40	<0.001[a]
**1F**	*glp-1* control	28.50 ± 1.51	0/40	
**Repeat 1**	*glp-1* 4 J/m^2	34.20 ± 1.83	0/41	0.0183[a]
	glp-1 40 J/m^2	35.40 ± 1.80	0/40	0.0126[a]
	glp-1 400 J/m^2	45.90 ± 1.19	0/40	<0.001[a]
**1F**	*glp-1* control	18.90 ± 1.11	1/41	
**Repeat 2**	*glp-1* 4 J/m2	21.60 ± 1.14	0/40	0.099[a]
	glp-1 40 J/m2	30.49 ± 1.34	0/37	<0.001[a]
	glp-1 400 J/m^2	43.50 ± 1.63	0/40	<0.001[a]
**1F**	*glp-1* control	22.74 ± 1.07	0/38	
**Repeat 3**	*glp-1* 4 J/m^2	26.40 ± 0.97	0/40	0.0175[a]
	glp-1 40 J/m^2	29.10 ± 1.11	0/40	<0.001[a]
	glp-1 400 j/m^2	39.60 ± 1.71	1/41	<0.001[a]
**1G Repeat 1**	*glp-1* intestine-specific control RNAi	5.00 ± 0.27	3/40	
	glp-1 intestine-specific *apn-1* RNAi	7.46 ± 0.41	0/41	<0.001[a]
	glp-1 intestine-specific *exo-3* RNAi	7.15 ± 0.44	0/40	<0.001[a]
**1G Repeat 2**	*glp-1* intestine-specific control RNAi	8.31 ± 0.42	0/32	
	glp-1 intestine-specific *apn-1* RNAi	10.35 ± 0.36	0/34	<0.001[a]
	glp-1 intestine-specific *exo-3* RNAi	10.11 ± 0.38	0/37	0.0014[a]
**1G Repeat 3**	*glp-1* intestine-specific control RNAi	10.75 ± 0.27	0/40	
	glp-1 intestine-specific *apn-1* RNAi	13.45 ± 0.19	1/40	<0.001[a]

	glp-1 intestine-specific *exo-3* RNAi	12.55 ± 0.31	0/41	<0.001[a]
**1H Repeat 1**	*glp-1* intestine-specific control RNAi	13.80 ± 0.68	0/40	
	glp-1 intestine-specific *apn-1* RNAi	24.60 ± 1.69	0/40	<0.001[a]
	glp-1 intestine-specific *exo-3* RNAi	18.71 ± 1.23	2/40	<0.001[a]
**1H Repeat 2**	*glp-1* intestine-specific control RNAi	14.05 ± 0.54	1/41	
	glp-1 intestine-specific *apn-1* RNAi	18.00 ± 1.20	1/41	0.0037[a]
	glp-1 intestine-specific *exo-3* RNAi	24.60 ± 1.69	0/40	<0.001[a]
**1H Repeat 3**	*glp-1* intestine-specific control RNAi	14.29 ± 0.73	0/42	
	glp-1 intestine-specific *apn-1* RNAi	20.10 ± 0.89	0/40	<0.001[a]
	glp-1 intestine-specific *exo-3* RNAi	21.60 ± 0.76	1/41	<0.001[a]
**2E**	*glp-1* control RNAi	20.80 ± 1.02	1/41	
**Repeat 1**	*glp-1* control RNAi UV	41.70 ± 1.47	0/40	<0.001[a]
	glp-1 ire-1 RNAi	15.60 ± 0.87	0/40	<0.001[a]
	glp-1 ire-1 RNAi UV	24.60 ± 1.53	0/40	<0.001[b]
**2E**	*glp-1* control RNAi	21.00 ± 1.02	0/40	
**Repeat 2**	*glp-1* control RNAi UV	40.11 ± 1.69	0/38	<0.001[a]
	glp-1 ire-1 RNAi	17.10 ± 1.11	0/40	0.015[a]
	glp-1 ire-1 RNAi UV	25.80 ± 1.38	1/40	<0.001[b]
**2E**	*glp-1* control RNAi	21.60 ± 0.97	1/40	
**Repeat 3**	*glp-1* control RNAi UV	38.40 ± 1.29	1/41	<0.001[a]
	glp-1 ire-1 RNAi	18.90 ± 1.03	0/40	0.064[a]
	glp-1 ire-1 RNAi UV	26.10 ± 1.40	0/40	<0.001[b]
**3B**	*glp-1*	20.70 ± 1.85	0/40	
**Repeat 1**	*glp-1; ced-3*	55.02 ± 4.03	0/41	<0.001[a]

	glp-1 UV	55.61 ± 3.81	4/41	<0.001[a]
	glp-1; *ced-3* UV	51.22 ± 4.22	2/41	<0.001[a]
**3B Repeat 2**	*glp-1*	12.92 ± 0.51	0/39	
	glp-1; *ced-3*	28.60 ± 2.28	0/40	<0.001[a]
	glp-1 UV	29.86 ± 1.76	0/42	<0.001[a]
	glp-1; *ced-3* UV	23.73 ± 1.76	0/41	<0.001[a]
**3B Repeat 3**	*glp-1*	14.63 ± 0.78	0/41	
	glp-1; *ced-3*	33.38 ± 2.69	0/32	<0.001[a]
	glp-1 UV	39.30 ± 2.61	0/40	<0.001[a]
	glp-1; *ced-3* UV	30.46 ± 2.34	0/39	<0.001[a]
**3C Repeat 1**	*glp-1*	16.00 ± 1.04	0/42	
	glp-1; *ced-4*	37.48 ± 3.69	7/41	<0.001[a]
	glp-1 UV	45.42 ± 3.63	2/41	<0.001[a]
	glp-1; *ced-4* UV	40.86 ± 2.74	0/42	<0.001[a]
**3C Repeat 2**	*glp-1*	14.63 ± 0.78	0/41	
	glp-1; *ced-4*	32.62 ± 2.65	0/39	<0.001[a]
	glp-1 UV	39.80 ± 2.60	1/41	<0.001[a]
	glp-1; *ced-4* UV	34.00 ± 2.81	0/36	<0.001[a]
**3C Repeat 3**	*glp-1*	19.61 ± 1.36	0/41	
	glp-1; *ced-4*	37.40 ± 2.17	0/43	<0.001[a]
	glp-1 UV	41.10 ± 2.16	0/40	<0.001[a]
	glp-1; *ced-4* UV	39.91 ± 1.92	0/43	<0.001[a]
**3D Repeat 1**	*glp-1* intestine-specific control RNAi	13.20 ± 0.84	0/40	
	glp-1 intestine-specific *ced-3* RNAi	19.20 ± 1.16	0/40	<0.001[a]
**3D Repeat 2**	*glp-1* intestine-specific control RNAi	13.00 ± 0.68	0/34	
	glp-1 intestine-specific *ced-3* RNAi	16.00 ± 1.15	0/34	0.038[a]
**3D Repeat 3**	*glp-1* intestine-specific control RNAi	13.20 ± 0.66	0/40	
	glp-1 intestine-specific *ced-3* RNAi	19.38 ± 1.86	0/36	0.002[a]

**4D**	*glp-1* control RNAi	12.88 ± 0.49	0/41	
**Repeat 1**	*glp-1 fat-6/7* RNAi	23.23 ± 2.62	0/38	<0.001[a]
	glp-1 control RNAi UV	33.29 ± 2.61	3/41	<0.001[a]
	glp-1 fat-6/7 RNAi UV	14.57 ± 0.76	0/42	<0.001[b]
**4D**	*glp-1* control RNAi	16.68 ± 1.54	0/41	
**Repeat 2**	*glp-1 fat-6/7* RNAi	25.43 ± 1.72	0/42	<0.001[a]
	glp-1 control RNAi UV	40.80 ± 2.24	0/45	<0.001[a]
	glp-1 fat-6/7 RNAi UV	20.29 ± 1.48	0/42	<0.001[b]
**4D**	*glp-1* control RNAi	14.93 ± 1.08	0/40	
**Repeat 3**	*glp-1 fat-6/7* RNAi	33.95 ± 2.55	0/37	<0.001[a]
	glp-1 control RNAi UV	36.28 ± 1.91	0/40	<0.001[a]
	glp-1 fat-6/7 RNAi UV	15.00 ± 0.78	0/42	<0.001[b]
**5D**	*glp-1* control RNAi	14.93 ± 1.08	0/41	
**Repeat 1**	*glp-1* control RNAi UV	36.86 ± 1.91	0/42	<0.001[a]
	glp-1 pmt-2 RNAi UV	18.00 ± 1.41	0/42	<0.001[b]
**5D**	*glp-1* control RNAi	15.19 ± 0.98	0/40	
**Repeat 2**	*glp-1* control RNAi UV	38.10 ± 2.51	0/40	<0.001[a]
	glp-1 pmt-2 RNAi UV	19.50 ± 1.04	0/40	<0.001[b]
**5D**	*glp-1* control RNAi	21.95 ± 1.23	0/41	
**Repeat 3**	*glp-1* control RNAi UV	40.85 ± 1.56	0/40	<0.001[a]
	glp-1 pmt-2 RNAi UV	27.00 ± 2.13	0/40	<0.001[b]
**5F**	*glp-1* control	15.41 ± 1.32	1/41	
**Repeat 1**	*glp-1* PC 18:1n9	24.34 ± 3.20	0/41	0.023[a]
	glp-1 control UV	40.48 ± 2.73	0/42	<0.001[a]
	glp-1 PC 18:1n9 UV	36.89 ± 2.41	2/41	0.302[b]
**5F**	*glp-1* control	13.64 ± 0.83	0/40	
**Repeat 2**	*glp-1* PC 18:1n9	27.63 ± 3.15	0/41	<0.001[a]

	glp-1 control UV	47.00 ± 3.81	2/40	<0.001[a]
	glp-1 PC 18:1n9 UV	39.24 ± 2.89	0/40	0.0966[b]
**5F**	*glp-1* control	16.00 ± 1.04	0/42	
**Repeat 3**	*glp-1* PC 18:1n9	28.63 ± 3.15	0/39	<0.001[a]
	glp-1 control UV	45.23 ± 3.65	0/43	<0.001[a]
	glp-1 PC 18:1n9 UV	38.10 ± 2.75	0/40	0.1132[b]
**5H**	*glp-1* control RNAi	30.90 ± 3.62	0/41	
**Repeat 1**	*glp-1*; *ced-3* control RNAi	50.29 ± 3.80	0/42	<0.001[a]
	glp-1; *ced-3* *pmt-2* RNAi	34.46 ± 3.67	2/41	0.012[d]
**5H**	*glp-1* control RNAi	37.71 ± 3.15	0/35	
**Repeat 2**	*glp-1*; *ced-3* control RNAi	59.10 ± 3.76	0/43	<0.001[a]
	glp-1; *ced-3* *pmt-2* RNAi	44.70 ± 3.55	0/40	<0.001[d]
**7E**	*glp-1*	29.85 ± 1.99	0/41	
**Repeat 1**	*glp-1*; *skn-1* gof	67.46 ± 4.75	0/37	<0.001[a]
	glp-1 UV	72.92 ± 2.71	4/41	<0.001[a]
	glp-1; *skn-1* gof UV	29.02 ± 2.17	0/43	<0.001[b]
**7E**	*glp-1* control	24.59 ± 0.58	0/41	
**Repeat 2**	*glp-1*; *skn-1* gof	61.54 ± 5.63	0/39	<0.001[a]
	glp-1 UV	64.19 ± 2.58	0/43	<0.001[a]
	glp-1; *skn-1* gof UV	29.40 ± 1.58	0/40	<0.001[b]
**7E**	*glp-1* control	21.95 ± 2.34	0/41	
**Repeat 3**	*glp-1*; *skn-1* gof	42.60 ± 3.39	0/40	<0.001[a]
	glp-1 UV	63.91 ± 3.05	0/43	<0.001[a]
	glp-1; *skn-1* gof UV	16.80 ± 1.52	0/40	<0.001[b]
**7F**	*glp-1*	12.62 ± 0.42	2/41	
**Repeat 1**	*glp-1*; *ced-3*	38.67 ± 3.53	7/41	<0.001[a]
	glp-1; *skn-1* gof	34.24 ± 2.40	0/41	<0.001[a]
	glp-1; *ced-3*;	25.54 ± 2.62	0/41	0.0115[d]

	skn-1 gof			
**7F**	*glp-1*	14.93 ± 1.00	1/40	
**Repeat 2**	*glp-1*; *ced-3*	38.77 ± 1.71	2/43	<0.001[a]
	glp-1; *skn-1* gof	33.73 ± 2.38	0/37	<0.001[a]
	glp-1; *ced-3*; *skn-1* gof	26.46 ± 2.05	0/39	0.0139[d]
**S1A**	*glp-1*	21.54 ± 0.62(days)	20/140	
**Repeat 1**	*glp-1* UV	15.37 ± 0.25(days)	28/140	<0.001[a]
**S1A**	*glp-1*	20.04 ± 1.03(days)	2/52	
**Repeat 2**	*glp-1* UV	15.21 ± 0.34(days)	4/60	<0.001[a]
**S1A**	*glp-1*	21.22 ± 0.95(days)	3/57	
**Repeat 3**	*glp-1* UV	15.36 ± 0.39(days)	4/60	<0.001[a]
**S1B**	*glp-4*	12.60 ± 0.41	0/40	
**Repeat 1**	*glp-4* UV	26.70 ± 1.61	0/42	<0.001[c]
**S1B**	*glp-4*	12.62 ± 0.42	0/39	
**Repeat 2**	*glp-4* UV	20.40 ± 1.14	0/40	<0.001[c]
**S1B**	*glp-4*	12.63 ± 0.41	0/40	
**Repeat 3**	*glp-4* UV	18.44 ± 1.02	0/41	<0.001[c]
**S1C**	*glp-4*	18.60 ± 0.94	0/40	
**Repeat 1**	*glp-4* UV	49.30 ± 1.85	1/40	<0.001[c]
**S1C**	*glp-4*	19.14 ± 0.97	0/37	
**Repeat 2**	*glp-4* UV	51.90 ± 1.66	0/40	<0.001[c]
**S1C**	*glp-4*	15.00 ± 0.82	0/40	
**Repeat 3**	*glp-4* UV	45.07 ± 1.48	0/41	<0.001[c]
**S1D**	*glp-4*	9.00 ± 0.31	0/36	
**Repeat 1**	*glp-4* UV	8.75 ± 0.30	0/36	0.5491[c]
**S1D**	*glp-4*	9.55 ± 0.43	0/38	
**Repeat 2**	*glp-4* UV	8.52 ± 0.43	0/41	0.1012[c]
**S1D**	*glp-4*	8.76 ± 0.26	0/38	
**Repeat 3**	*glp-4* UV	8.93 ± 0.27	0/40	0.6488[c]
**S1E**	*glp-4*	10.95 ± 0.29	0/40	
**Repeat 1**	*glp-4* UV	10.20 ± 0.46	0/40	0.9671[c]
**S1E**	*glp-4*	10.92 ± 0.27	0/39	

**Repeat 2**	*glp-4* UV	10.69 ± 0.42	0/39	0.6476[c]
**S1E**	*glp-4*	11.40 ± 0.27	0/40	
**Repeat 3**	*glp-4* UV	11.4 ± 0.41	0/40	0.174[c]
**S1F**	WT	29.84 ± 1.69	0/37	
**Repeat 1**	WT UV	52.31 ± 3.12	0/39	<0.001[a]
**S1F**	WT	29.65 ± 2.01	0/34	
**Repeat 2**	WT UV	46.80 ± 2.39	0/40	<0.001[a]
**S1F**	WT	15.43 ± 1.42	0/35	
**Repeat 3**	WT UV	39.22 ± 2.27	0/41	<0.001[a]
**S1G**	WT	11.08 ± 1.04	1/42	
**Repeat 1**	WT UV	20.25 ± 1.92	1/42	<0.001[a]
**S1G**	WT	17.21 ± 1.30	0/41	
**Repeat 2**	WT UV	22.88 ± 1.25	0/43	0.0029[a]
**S1G**	WT	17.40 ± 1.03	0/40	
**Repeat 3**	WT UV	23.70 ± 1.47	0/40	<0.001[a]
**S1H**	WT	7.85 ± 0.20	0/40	
**Repeat 1**	WT UV	9.01 ± 0.22	2/40	<0.001[a]
**S1H**	WT	5.83 ± 0.27	0/35	
**Repeat 2**	WT UV	6.51 ± 0.29	0/39	0.089[a]
**S1H**	WT	7.35 ± 0.15	0/37	
**Repeat 3**	WT UV	7.88 ± 0.15	0/33	0.0186[a]
**S1I**	WT	6.70 ± 0.27	0/37	
**Repeat 1**	WT UV	7.85 ± 0.30	2/37	0.004[a]
**S1I**	WT	7.85 ± 0.20	0/40	
**Repeat 2**	WT UV	9.03 ± 0.22	0/39	0.0002[a]
**S1I**	WT	9.23 ± 0.30	0/39	
**Repeat 3**	WT UV	10.18 ± 0.33	0/38	0.0229[a]
**S1J Repeat 1**	*glp-1* intestine-specific control RNAi	7.17 ± 0.33	0/41	
	glp-1 intestine-specific *apn-1* RNAi	8.85 ± 0.26	0/40	0.001[a]
	glp-1 intestine-specific *exo-3* RNAi	8.35 ± 0.34	0/40	0.017[a]

**S1J Repeat 2**	*glp-1* intestine-specific control RNAi	8.45 ± 0.34	0/40	
	glp-1 intestine-specific *apn-1* RNAi	11.54 ± 0.27	0/39	<0.001[a]
	glp-1 intestine-specific *exo-3* RNAi	11.00 ± 0.31	0/38	<0.001[a]
**S1J Repeat 3**	*glp-1* intestine-specific control RNAi	7.17 ± 0.33	0/41	
	glp-1 intestine-specific *apn-1* RNAi	8.35 ± 0.34	0/40	0.0172[a]
	glp-1 intestine-specific *exo-3* RNAi	8.85 ± 0.26	0/40	0.0011[a]
**S1K Repeat 1**	*glp-1* intestine-specific control RNAi	12.08 ± 0.34	0/40	
	glp-1 intestine-specific *apn-1* RNAi	12.15 ± 0.33	1/41	0.905[a]
	glp-1 intestine-specific *exo-3* RNAi	12.30 ± 0.33	0/40	0.674[a]
**S1K Repeat 1**	*glp-1* intestine-specific control RNAi	14.78 ± 0.94	0/40	
	glp-1 intestine-specific *apn-1* RNAi	14.77 ± 0.94	0/40	0.9184[a]
	glp-1 intestine-specific *exo-3* RNAi	14.63 ± 0.93	2/42	0.9655[a]
**S1K Repeat 1**	*glp-1* intestine-specific control RNAi	11.55 ± 0.38	0/40	
	glp-1 intestine-specific *apn-1* RNAi	11.85 ± 0.49	0/40	0.8141[a]
	glp-1 intestine-specific *exo-3* RNAi	11.63 ± 0.37	1/41	0.8126[a]
**S3A**	*glp-1*	12.29 ± 0.29	0/41	
**Repeat 1**	*glp-1*; *ced-3*	19.89 ± 1.81	6/40	<0.001[a]
**S3A**	*glp-1*	12.34 ± 0.34	0/35	
**Repeat 2**	*glp-1*; *ced-3*	22.18 ± 2.73	0/33	<0.001[a]
**S3A**	*glp-1*	12.36 ± 0.36	0/33	
**Repeat 3**	*glp-1*; *ced-3*	26.00 ± 3.32	0/36	<0.001[a]
**S3B**	*glp-1*	11.93 ± 0.36	0/42	
**Repeat 1**	*glp-1*; *ced-3*	11.78 ± 0.43	2/40	0.951[a]

**S3B**	*glp-1*	10.45 ± 0.47	0/40	
**Repeat 2**	*glp-1*; *ced-3*	10.15 ± 0.50	0/40	0.791[a]
**S3B**	*glp-1*	10.25 ± 0.42	0/40	
**Repeat 3**	*glp-1*; *ced-3*	9.90 ± 0.35	0/40	0.2735[a]
**S3C**	*glp-1*	9.07 ± 0.32	0/41	
**Repeat 1**	*glp-1*; *ced-3*	9.05 ± 0.34	0/40	0.957[a]
**S3C**	*glp-1*	8.05 ± 0.31	0/41	
**Repeat 2**	*glp-1*; *ced-3*	7.95 ± 0.37	0/41	0.9012[a]
**S3C**	*glp-1*	8.78 ± 0.29	0/41	
**Repeat 3**	*glp-1*; *ced-3*	9.03 ± 0.29	0/39	0.5729[a]
**S3D**	*glp-1* control RNAi	28.50 ± 1.32	0/40	
**Repeat 1**	*glp-1 egl-1* RNAi	39.00 ± 1.93	0/42	<0.001[a]
	glp-1 control RNAi UV	43.71 ± 1.45	0/40	<0.001[a]
	*glp-1 egl-1*RNAi UV	44.31 ± 1.58	0/39	0.6621[b]
**S3D**	*glp-1* control RNAi	29.14 ± 1.83	0/35	
**Repeat 2**	*glp-1 egl-1* RNAi	44.70 ± 1.59	0/40	<0.001[a]
	glp-1 control RNAi UV	44.23 ± 1.59	0/35	<0.001[a]
	glp-1 egl-1 RNAi UV	45.71 ± 1.35	0/42	0.794[b]
**S3E**	*glp-1*	15.60 ± 0.87	0/40	
**Repeat 1**	*glp-1*; *ced-13*	14.70 ± 0.79	0/41	0.4487[a]
	glp-1 UV	43.80 ± 1.38	0/40	<0.001[a]
	glp-1; *ced-13* UV	42.00 ± 1.27	0/40	0.3016[b]
**S3E**	*glp-1*	14.40 ± 0.76	0/40	
**Repeat 2**	*glp-1*; *ced-13*	17.40 ± 0.94	0/41	0.017[a]
	glp-1 UV	44.40 ± 1.60	0/39	<0.001[a]
	glp-1; *ced-13* UV	43.80 ± 1.24	0/40	0.500[b]
**S5E**	*glp-1* control	16.32 ± 1.56	2/42	
**Repeat 1**	*glp-1* PC 16:0	17.52 ± 1.94	0/42	0.725[a]
	glp-1 PC 18:0	19.90 ± 2.19	0/41	0.227[a]
	glp-1 control UV	38.78 ± 2.52	3/42	<0.001[a]

	glp-1 PC 16:0 UV	45.07 ± 2.77	1/42	0.519[b]
	glp-1 PC 18:0 UV	41.05 ± 2.59	0/42	0.274[b]
**S5E**	*glp-1* control	13.80 ± 0.68	2/40	
**Repeat 2**	*glp-1* PC 16:0	14.10 ± 0.84	0/40	0.7664[a]
	glp-1 PC 18:0	15.00 ± 1.02	0/41	0.3121[a]
	glp-1 control UV	47.70 ± 1.97	0/42	<0.001[a]
	glp-1 PC 16:0 UV	45.90 ± 1.79	1/42	0.3298[b]
	glp-1 PC 18:0 UV	45.60 ± 1.42	0/42	0.5865[b]
**S5E**	*glp-1* control	14.40 ± 0.76	0/40	
**Repeat 3**	*glp-1* PC 16:0	13.20 ± 0.71	0/40	0.2716[a]
	glp-1 PC 18:0	13.50 ± 0.76	0/41	0.4259[a]
	glp-1 control UV	47.10 ± 1.66	0/42	<0.001[a]
	glp-1 PC 16:0 UV	47.40 ± 1.75	0/42	0.8047[b]
	glp-1 PC 18:0 UV	44.70 ± 1.80	0/42	0.2851[b]
**S5F Repeat 1**	*glp-1* control RNAi UV	42.79 ± 2.13	1/41	
	glp-1 pmt-2 RNAi UV	25.17 ± 0.81	1/41	<0.001[a]
	glp-1 control RNAi UV with PC 18:1n9	39.80 ± 2.70	0/41	0.131[a]
	glp-1 pmt-2 RNAi UV with PC 18:1n9	38.05 ± 2.19	0/41	0.542[e]
**S5F Repeat 2**	*glp-1* control RNAi UV	42.60 ± 1.27	0/40	
	glp-1 pmt-2 RNAi UV	15.00 ± 0.82	0/40	<0.001[a]
	glp-1 control RNAi UV with PC 18:1n9	43.50 ± 1.57	1/41	0.5115[a]
	glp-1 pmt-2 RNAi UV with PC 18:1n9	39.60 ± 1.54	0/40	0.2155[e]
**S5F Repeat 3**	*glp-1* control RNAi UV	43.08 ± 2.16	0/39	
	glp-1 pmt-2 RNAi UV	24.59 ± 0.58	0/41	<0.001[a]
	glp-1 control RNAi UV with PC 18:1n9	39.80 ± 2.70	0/41	0.4447[a]
	glp-1 pmt-2 RNAi UV with PC 18:1n9	38.05 ± 2.19	0/41	0.1164[e]
**S6E**	*glp-1*	20.16 ± 1.38	0/50	

**Repeat 1**	*glp-1; skn-1* lof	15.51 ±0.95	0/42	0.0068[a]
	glp-1 UV	36.00 ±1.76	0/41	<0.001[a]
	glp-1; skn-1 lof UV	34.60 ±1.68	0/43	0.5085[b]
**S6E**	*glp-1*	18.77 ±1.13	0/39	
**Repeat 2**	*glp-1; skn-1* lof	16.80 ±0.93	0/40	0.1815[a]
	glp-1 UV	46.80 ±2.54	0/40	<0.001[a]
	glp-1; skn-1 lof UV	45.00 ±1.79	0/40	0.1014[b]
**S6F Repeat 1**	*glp-1*; *skn-1* gof intestine-specific control RNAi	9.55 ±0.35	0/40	
	glp-1; *skn-1* gof intestine-specific *apn-1* RNAi	9.88 ±0.36	0/41	0.220[a]
	glp-1; *skn-1* gof intestine-specific *exo-3* RNAi	9.83 ±0.42	0/40	0.336[a]
**S6F Repeat 2**	*glp-1*; *skn-1* gof intestine-specific control RNAi	5.71 ±0.22	0/41	
	glp-1; *skn-1* gof intestine-specific *apn-1* RNAi	6.25 ±0.29	0/40	0.242[a]
	glp-1; *skn-1* gof intestine-specific *exo-3* RNAi	6.16 ±0.34	0/38	0.121[a]

[a] vs WT(*glp-1*)/control RNAi

[b] vs *glp-1* UV/ control RNAi UV

[c] vs *glp-4*

[d] vs *glp-1*; *ced-3*

[e] vs *glp-1* control RNAi UV with PC 18:1n9
